# High-*k* water intermediate layer-mediated charge transfer modulation toward stable and augmented output generation of droplet-based electricity generators

**DOI:** 10.1038/s41598-025-27319-9

**Published:** 2025-12-30

**Authors:** Sunmin Jang, Soban Ali Shah, Dong Yong Park, Sumin Cho, Dongik Kam, Girak Gwon, Yoonsang Ra, Hee Jae Hwang, Moonwoo La, Sung Jea Park, Dongwhi Choi

**Affiliations:** 1 Department of Mechanical Engineering (Integrated Engineering Program), Kyung Hee University, 1732 Deogyeong-daero, Yongin, 17104 Gyeonggi Republic of Korea; 2 Advanced Mobility Components Group, Korea Institute of Industrial Technology, 320 Techno sunhwan-ro, Yuga-eup, Dalsung-gun Republic of Korea; 3 School of Mechanical Engineering, Chonnam National University, 77 Yongbong-ro, Buk-gu, Gwangju, 61186 Republic of Korea; 4 School of Mechanical System Engineering, Kumoh National Institute of Technology, 61, Daehak-ro, Gumi-si, 39177 Gyeongsangbuk-do Republic of Korea; 5 School of Mechanical Engineering, Korea University of Technology and Education, 1600 Chungjeol-ro, Cheonan, 31253 Chungnam Republic of Korea; 6 Advanced Technology Research Centre, Korea University of Technology and Education, 1600 Chungjeol-ro, Cheonan, 31253 Chungnam Republic of Korea; 7 Future Convergence Engineering, Korea University of Technology and Education, 1600 Chungjeol-ro, Cheonan, 31253 Chungnam Republic of Korea

**Keywords:** Energy science and technology, Engineering

## Abstract

Droplet-based electricity generators (DEGs), which share structural analogies with field-effect transistors (FETs), have garnered increasing attention for droplet energy harvesting. DEGs typically exhibit sharp output peaks with short charge transfer durations, which need to be extended to enhance the root mean square (RMS) output for practical use in energy harvesting. To address this limitation, this study introduces a methodology for spontaneously forming a high-*k* water intermediate (WIn) layer by capturing working droplets, inspired by strategies for FET. Based on the operation mechanism of the DEG, this strategy yields both an increase in the amount of transferred charge and an extension in charge transfer duration, thereby augmenting current output. To further demonstrate its significance, RMS output enhancement is validated under conditions with similar peak output levels but an augmented charge transfer process, highlighting its practical advantage. In addition, the extended charge transfer duration enables sporadic peak overlapping, potentially resulting in a direct current (DC)-like output generation. This behavior contributes to RMS output enhancement, thereby improving the energy harvesting capacity. This study underscores the importance of the charge transfer process augmentation via the spontaneously generated high-*k* WIn layer, paving the way for the practical implementation of DEGs.

**Supplementary Information:**

The online version contains supplementary material available at 10.1038/s41598-025-27319-9.

## Introduction

With the recent energy crisis, various energy harvesting platforms that can scavenge abandoned energy are gaining attention as stepping stones toward its alleviation^1–5^. Among abandoned energy sources, water is considered a promising energy source for harvesting because of its ubiquitous presence on Earth^6–8^. Recently, researchers have focused on harvesting energy from raindrops because of their considerable potential, and energy harvesting platforms such as piezoelectric^9,10^, hydrovoltaic^11,12^, and triboelectric nanogenerators^13–15^ have been proposed for droplet energy harvesting. In 2020, a droplet-based electricity generator (DEG), a liquid–solid contact electrification-based droplet energy harvesting platform, was proposed^16^. With its field-effect transistor (FET)-inspired structure consisting of a dielectric contact layer between two disparate electrodes, a sharp surging peak in the electrical output with a short charge transfer duration of several milliseconds can be generated by droplets impinging on the DEG^17–19^. This high electrical output generation behavior enables DEG utilization in various practical applications, such as power sources for electric devices^20–22^, cathodic protection^23^, and smart windows^24,25^. Various studies have focused on enhancing the amplitude of the electrical output from a DEG^26–30^. However, for utilization as a power source, the root mean square (RMS) value of the electrical output must be considered, which is equivalent to DC electricity^31^. Considering that the sharp surging peak resulting from the short charge transfer duration of the DEG likely results in a decrease in the RMS electrical output, the charge transfer duration of the DEG must be extended for effective energy harvesting^32^. According to previous studies, the charge transfer behavior of the DEG follows the discharging behavior of the RC circuit, such that the load resistance applied to the DEG, the resistance of the working droplet, and the capacitance of the dielectric contact layer affect the duration of charge transfer^33–35^. However, when considering the definition of the current, the amount of transferred charge during a unit of time, a simple extension of the charge transfer duration potentially decreases the amplitude of the electrical output. Thus, augmentation of the electrical output by increasing not only the charge transfer duration but also the amount of transferred charge must be considered simultaneously.

Considering the structural analogy between DEG and FET, as mentioned above, the conventional strategy suggested for FET performance improvement can be a candidate for clear a hurdle which DEG technology faces. The FET is composed of a source, gate, drain, and an insulating layer between the gate and substrate. When a voltage is applied to the gate, charge carriers are attracted and generate a conductive channel between the source and drain^36,37^. In this context, various studies have focused on utilizing a high dielectric constant ($\:k$) material in the insulating layer to encourage conductive channel generation under a lower threshold voltage and generate a highly conductive channel under the same gate voltage without current leakage and dielectric breakdown, which are fatal for its operation^38,39^. Similarly, the DEG is operated with liquid–solid contact electrification between the droplet (gate) and the dielectric contact layer, resulting in charge induction and transfer between the bottom electrode (source) and the top electrode (drain)^40,41^. By this analogy, utilizing a high-$\:k$ material for the dielectric contact layer of the DEG possibly promotes charge induction across the dielectric contact layer and electrode, thereby enhancing the amount of transferred charge^42,43^. In addition, considering the charge transfer behavior of the DEG, applying a high-$\:k$ material to the DEG can be a novel strategy for effective droplet energy harvesting with an enhanced amount of charge transfer and extended charge transfer duration during its operation at the same time.

In this study, water, a high-$\:k$ dielectric, is applied as the dielectric contact layer of a DEG, and the relationship between its application and charge transfer behavior as well as electrical output is discovered. Water is utilized as an intermediate layer embedded between the dielectric contact layer, which is advantageous for generating electric charge through liquid–solid contact electrification, and the bottom electrode to garner its advantageous aspect. Based on the structural analogy with FETs, in the presence of a water intermediate (WIn) layer, a larger amount of charge can be induced on the bottom electrode from the charges developed on the dielectric contact layer by liquid–solid contact electrification, which can directly enhance the amount of transferred charge during its operation. Simultaneously, the charge transfer duration can be further extended according to the operating mechanism of the DEG; thus, an augmented electrical output can be generated and stably maintained for an extended duration. Considering the droplet impact is necessary for the DEG operation, this WIn layer can be spontaneously developed with continuous droplet introduction, which allows generation of the high-$\:k$ intermediate layer which is advantageous for electrical output augmentation without cumbersome process. Moreover, these enhancements in the amount of transferred charge and the extension of the charge transfer duration can be effective in practice, not only in the measured output. In this context, to show the necessity of this charge transfer process analysis, the differences in two cases that possess the same electrical output with different amounts of transferred charge and charge transfer durations re compared by calculating the RMS value of the electrical output and charging capacitor. Although the amplitude of the electrical output is similar, a higher RMS output can be generated, and the capacitor can be largely charged in the case of a larger amount of transferred charge and longer charge transfer duration, which emphasizes its significance. Furthermore, especially with the extension of the charge transfer duration with the application of the water intermediate layer, there is a higher possibility for the two peaks to overlap sporadically, which can possibly lead to output generation in the DC. With this peak overlap, the interval between the two peaks disappears; thus, the RMS output can be further increased, which demonstrates its higher effectiveness for wide practical application as a power source^44–46^. In this context, this study highlights the necessity of focusing on the charge transfer process during the DEG operation to evaluate its practical effectiveness.

## Results and discussion

### High-$\:k$ intermediate layer application strategy on the DEG

**Fig. 1:**
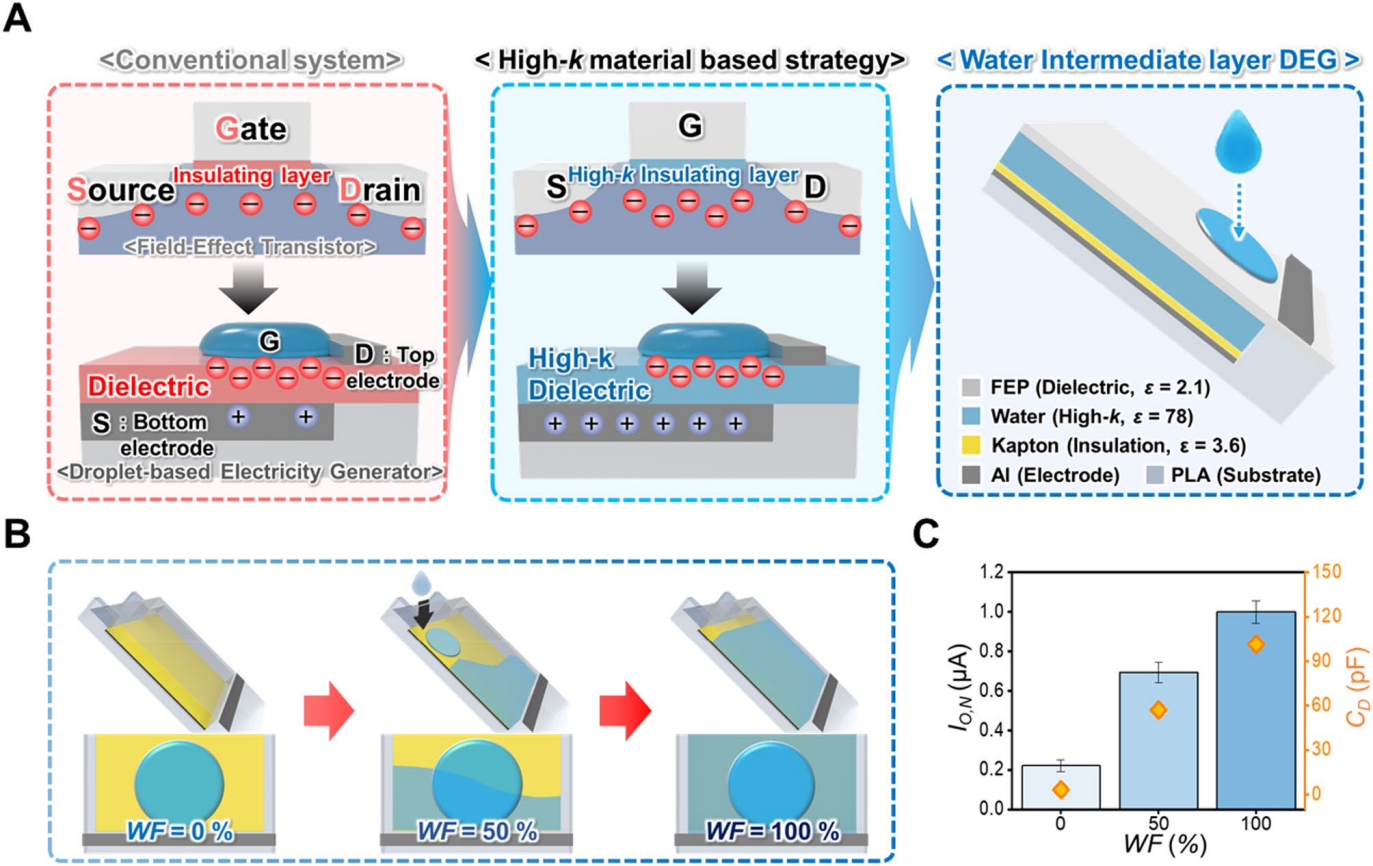
(**A**) Structural advancement and overall systematic composition of WIn-DEG. (**B**) Schematic of the WIn layer development and (**C**) current output generation behavior and capacitance-changing behavior during WIn layer development.

As illustrated in Fig. 1A, the FET and DEG have structural analogies, which both comprise gate, source, drain, and insulating layers. In an FET, the voltage applied to the gate attracts electrons, generating a conductive channel between the source and drain. In the DEG, the droplet functions similar to that of the gate (G), whereas the bottom and top electrodes act as the source (S) and drain (D), respectively. During FET operation, the capacitance of the insulating layer is a crucial factor because electron attraction is significantly affected by the electric field resulting from the gate voltage. In this context, enhancing the capacitance of the insulating layer can promote the generation of a conductive channel, allowing the FET to operate at a lower threshold voltage and generate a channel with higher conductivity under the same gate voltage. In this context, reducing the thickness of the insulating layer is considered to increase capacitance. However, there is a definite limitation on the thickness decrement, and an excessive thickness decrement may result in a dielectric breakdown, which is critical for its operation. Thus, the utilization of high-$\:k$ materials is highlighted by the achievement of a larger electric field with the same gate voltage, thereby attracting more electrons while circumventing the disadvantages resulting from excessive layer thinning. Similarly, the capacitance of the dielectric contact layer for the DEG is critical for its operation because it is closely related to the amount of charge induced on the bottom electrode (source) from the surface charge resulting from liquid–solid contact electrification. Because the amount of induced charge significantly affects the electrical output of the DEG, the utilization of high-$\:k$ materials for the dielectric layer can be considered a promising strategy for enhancing energy harvesting efficiency.

For the facile introduction of high-$\:k$ materials to the DEG, water, which is a commonly encountered high-$\:k$ material, is applied to the high-$\:k$ dielectric contact layer. However, given the operation mechanism of the DEG, not only the charge induced on the electrode but also the surface charging on the dielectric contact layer must be considered. In this context, the direct utilization of water as a dielectric contact layer can inhibit contact electrification; thus, embedding a water layer between the dielectric contact layer and the electrode should be a better strategy to simultaneously provide larger surface charging and charge induction at the same time. Considering the superiority of fluoropolymers in the liquid-solid contact electrification—due to their hydrophobic wetting property and high electron affinity from the fluorinated groups on their surfaces—the fluorinated ethylene propylene (FEP) is utilized as a dielectric contact layer to guarantee electrical output generation^47,48^. This FEP film is attached to a polylactic acid (PLA) substrate so that a WIn layer can be generated through a continuous droplet impinging underneath the FEP film. This implies that a high-$\:k$ intermediate layer can be spontaneously generated without a complex fabrication process, given that droplet impingement is necessary for DEG operation. Considering that the WIn layer has sufficient electrical resistance to act as the electrode of the DEG^49^, an additional insulation layer is introduced between the WIn layer and the bottom electrode to prevent their direct contact. In addition, the top electrode is attached to the surface of the FEP, allowing direct droplet impingement on the WIn layer-applied DEG (WIn-DEG) to generate an electrical output.

Given the spontaneous generation of the WIn layer from consistent droplet impingement, as shown in Fig. 1B and Supporting Video 1, the current generation behavior during WIn layer development is shown in Fig. 1C. To quantify the effect of the WIn layer-filling process on the electrical output, the water-filling rate (*WF*) is quantified by considering the proportion of the intermediate layer between air and water. Because the DEG operates with droplet impingement and its effective region is primarily related to the surface area of the droplet in contact with the top electrode, the WIn layer filling half of the droplet surface area is defined as *WF* of 50%, as shown in Fig. 1B. As the *WF* increases from 0% to 100%, the normalized current output ($\:{I}_{O,N}$) from the WIn-DEG under the impinging DI water droplet with a volume of 40 µL increases from 0.22 ± 0.03 to 1.00 ± 0.06, as shown in Fig. 1C. This behavior can be attributed to the filling of the air gap with DI water, which has a higher $\:k$ value. To verify this phenomenon, the total capacitance of the dielectric, intermediate, and insulation layer ($\:{C}_{D}$) is measured, showing an increase from 3.24 pF to 101.55 pF at a frequency of 1000 Hz as the *WF* increased from 0% to 100%. Based on these experimental results, the $\:{I}_{O,N}$ directly follows the behavior of the $\:{C}_{D}$ under various *WF*s, which highlights the importance of capacitance on the amplitude of the electrical output of the DEG. Given that the strong dependence on the *WF* of the WIn layer, its evaporation rate is monitored for 35 h under both room condition and oven condition by measuring the mass of the WIn layer ($\:{M}_{WIn}$) as shown in Figure S1. Under the high-temperature and low-humidity environment of the oven condition, approximately 86.54% of the WIn layer remained after 35 h, whereas 96.63% remained under room conditions. In this context, a long-term operational pause of the WIn-DEG can cause a temporary degradation in output at the beginning of the operation; however, since the WIn layer can be spontaneously regenerated upon droplet introduction, the current output can be fully restored after several droplet inputs.

### Theoretical modeling of the charge transfer process during DEG operation

**Fig. 2:**
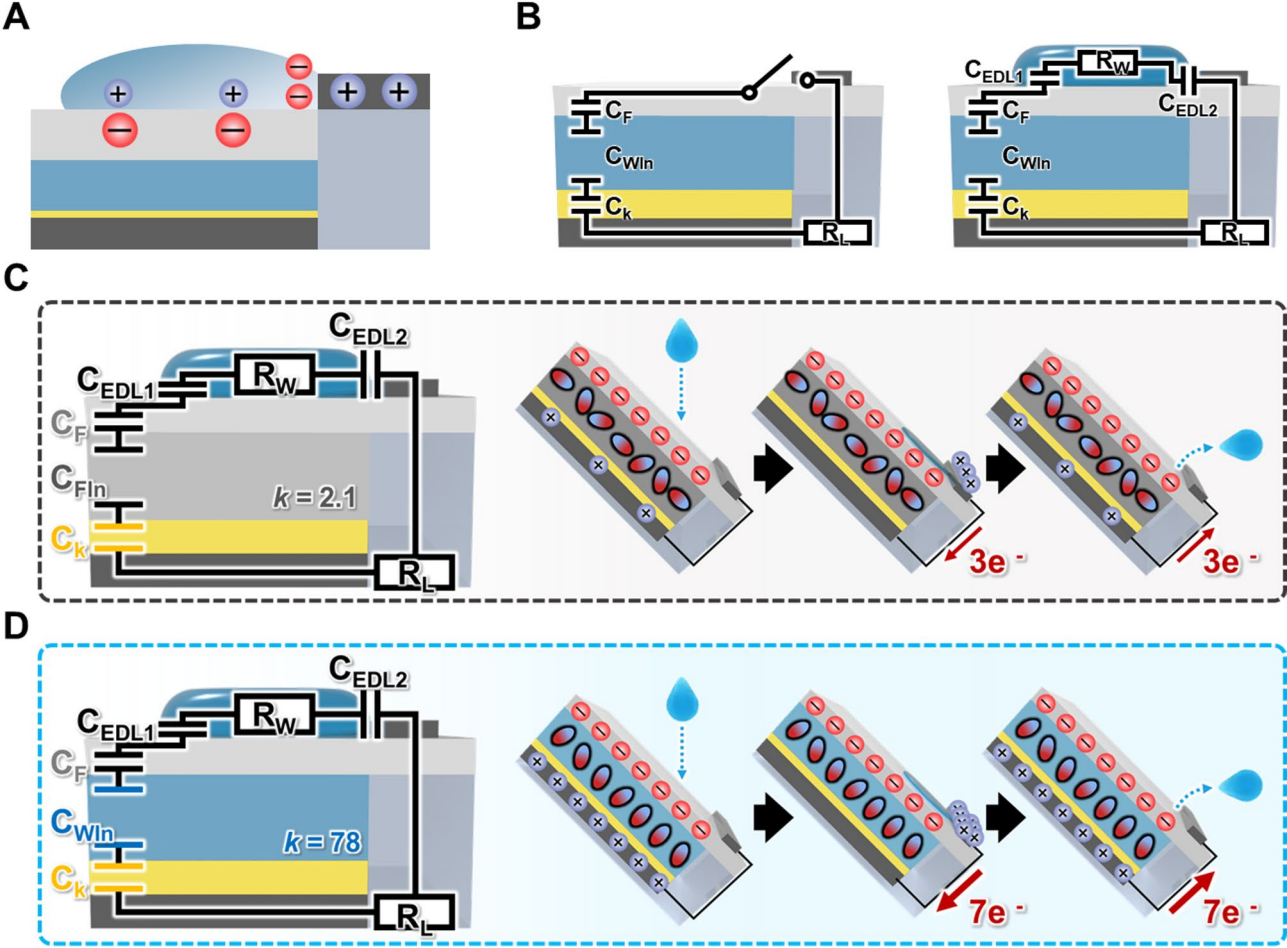
(**A**) Charge development behavior upon droplet contact. (**B**) Equivalent circuit model of the WIn-DEG during its operation. Schematic of the equivalent circuit model and charge transfer behavior of (**C**) FIn-DEG and (**D**) WIn-DEG.

The electrical output generation mechanism and theoretical model of the WIn-DEG are shown in Fig. 2. The DEG generates electrical output mainly through two interactions: contact electrification between the droplet and the dielectric contact layer (interfacial effect) and electric double layer (EDL) generation between the droplet and the top electrode, which results from the contact between the droplet and the top electrode during its impact on the dielectric contact layer (bulk effect)^16^. The FEP tends to be negatively charged after continuous contact electrification between the liquid and solid^50–52^, according to the theoretical basis elaborated in the Supporting Information and Figure S2. Accordingly, to maintain electroneutrality, positive charges are induced on the bottom electrode. The presence of a high-$\:k$ water intermediate layer may allow the induction of a positive charge similar to the amount of negative surface charge. As the droplet impinges on the dielectric contact layer, the positive charges in the droplet are spontaneously attracted toward the negative charges from contact electrification, whereas the negative charges in the droplet are repelled from the dielectric contact layer. After the droplet spreads and touches the top electrode, negative charges are attracted toward the top electrode. However, this charge-pair arrangement destroys electroneutrality because the electric field resulting from the surface charge is neutralized. To recover its electroneutrality, electrons on the top electrode migrate from the top electrode to the bottom electrode, resulting in a sharp surge in the electrical output peak. It is inferred that the top electrode becomes positively charged so that the EDL is generated at the interface between the droplet and the top electrode, which satisfies electroneutrality, as shown in Fig. 2A. To clarify the electrical output generation mechanism of the DEG, an equivalent circuit model of the WIn–DEG operation is shown in Fig. 2B. Before the droplet introduction, the equivalent circuit remains open, while only comprising the capacitor model of $\:{C}_{D}$, which comprises the Kapton layer ($\:{C}_{k}$), water intermediate layer ($\:{C}_{WIn}$), FEP layer ($\:{C}_{F}$), and potential load resistance from the wire connection ($\:{R}_{L}$). As the droplet impinges and contacts the FEP surface and top electrode simultaneously, the circuit is closed, and a new capacitor model of the EDL between the droplet and FEP ($\:{C}_{EDL1}$), the EDL between the droplet and top electrode ($\:{C}_{EDL2}$), and a resistor model in the droplet ($\:{R}_{W}$) emerge newly. Because the thicknesses of the $\:{C}_{k}$, $\:{C}_{WIn}$, and $\:{C}_{F}$ are considered to be much larger than those of the $\:{C}_{EDL1}$ and $\:{C}_{EDL2}$, they can be considered as charge sources that can be discharged under the closed-loop circuit following the discharging behavior of the RC circuit.

Based on this model, the FEP intermediate layer applied DEG (FIn-DEG) is fabricated as a comparative system to further investigate the effect of the capacitance of the DEG on its charge transferring process and electrical output generation, as shown in Fig. 2C. For an accurate comparison, the dimensions of the FEP intermediate layer are fabricated to be equal to those of WIn-DEG. Compared to WIn-DEG, the capacitance of the FEP intermediate layer ($\:{C}_{FIn}$) is lower than $\:{C}_{WIn}$, because the $\:k$ of FEP (2.1) is lower than that of water (78.4)^53,54^. This results in a decrease in the overall capacitances of the $\:{C}_{k}$, $\:{C}_{FIn}$, and $\:{C}_{F}$, such that the net charge generated on the FEP dielectric contact cannot fully polarize the FEP intermediate (FIn) layer; thus, only a few charges can be induced on the bottom electrode. This probably resulted in a decrease in the transferred charge during the operation of the FIn-DEG. However, considering the high $\:k$ value of water, the WIn layer can be largely polarized by the negative charge generated on the dielectric contact layer; thus, a larger amount of charge can be induced on the bottom electrode, as shown in Fig. 2D. In this regard, a larger amount of charge can be transferred during the operation of the WIn-DEG compared with the FIn-DEG.

According to previous studies, both the amount of transferred charge and the charge transfer duration during its operation are affected by the $\:k$ of the intermediate layer, as the DEG operation is related to the discharge of the capacitor model of the insulating layer, intermediate layer, and dielectric contact layer, which is considered the charge source^33^. In this context, the charge transfer duration ($\:\tau\:$) can be demonstrated as follows:

1 $$\:\tau\:={C}_{D}\times\:\left({R}_{L}+{R}_{W}\right)=\left(\frac{1}{\raisebox{1ex}{$1$}\!\left/\:\!\raisebox{-1ex}{${C}_{F}$}\right.+\raisebox{1ex}{$1$}\!\left/\:\!\raisebox{-1ex}{${C}_{In}$}\right.+\raisebox{1ex}{$1$}\!\left/\:\!\raisebox{-1ex}{${C}_{K}$}\right.}\right)\times\:({R}_{L}+{R}_{W})$$

where $\:{C}_{In}$ denotes the capacitance of the intermediate layer. In particular, as the thickness of the intermediate layer is larger than that of the dielectric contact layer and insulating layer, tailoring the capacitance of the intermediate layer may affect its operation more. Thus, Eq. 1 can be simplified as follows:

2 $$\:\tau\:={C}_{In}\times\:({R}_{L}+{R}_{W})$$

With this theoretical model, it can be inferred that the high-$\:k$ intermediate layer can affect both the amount of transferred charge and the charge transfer duration, which can significantly contribute to the augmentation of the current output that allows effective electricity harvesting with the DEG.

### Verification of the theoretical modeling

**Fig. 3:**
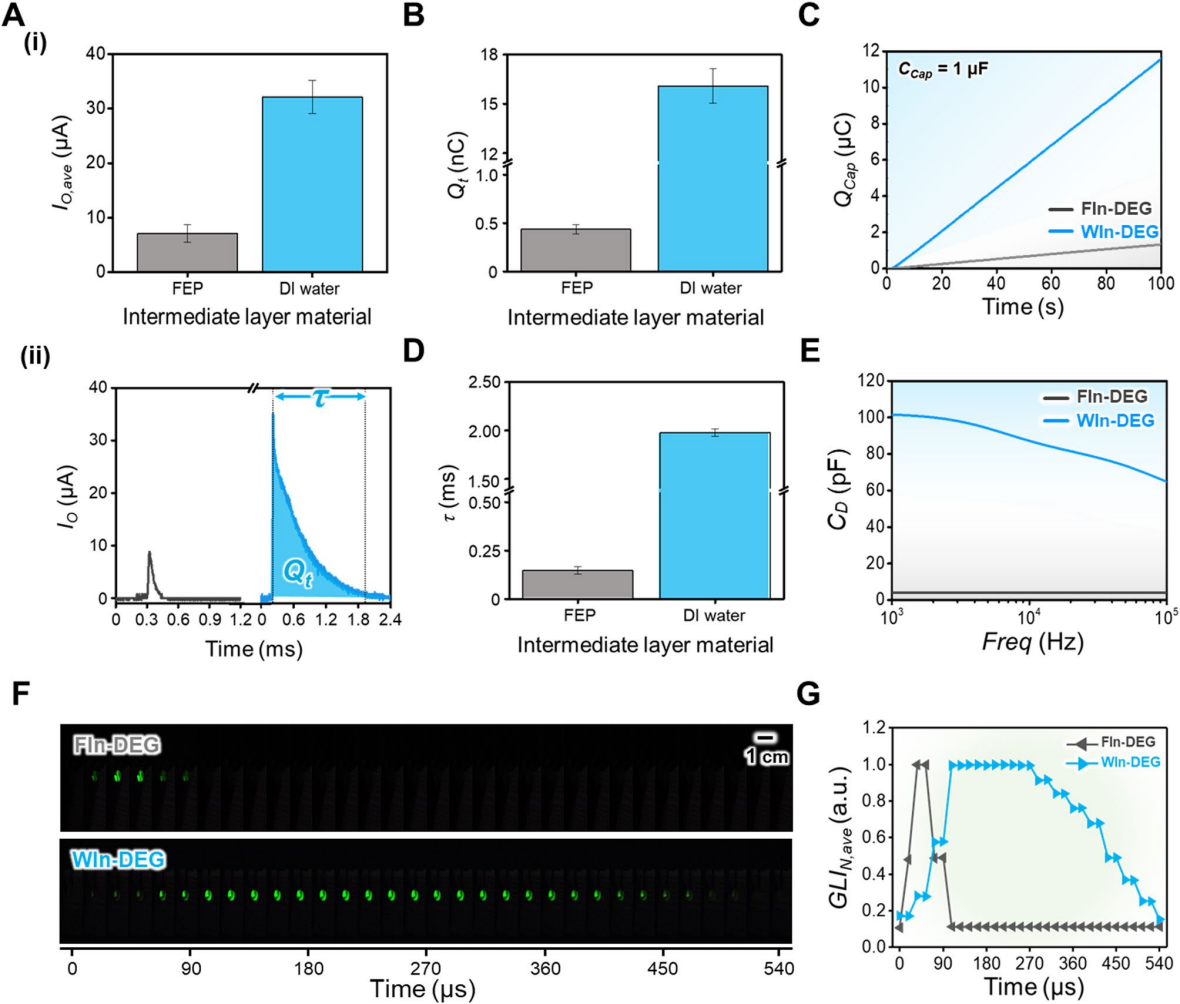
(**A**) **(i)** Average current output ($\:{I}_{O,ave}$) generated from FIn-DEG and WIn-DEG, and **(ii)** representative peak of the current output for the explanation of the amount of transferred charge ($\:{Q}_{t}$) and charge transfer duration ($\:\tau\:$). (**B**) $\:{Q}_{t}$ of FIn-DEG and WIn-DEG and (**C**) capacitor charging behavior for the verification of the difference in $\:{Q}_{t}$. (**D**) $\:\tau\:$ of FIn-DEG and WIn-DEG and (**E**) capacitance of the dielectric contact layer for the verification of the difference in $\:\tau\:$. (**F**) LED illumination behavior under the FIn-DEG and WIn-DEG operation captured from Supporting Video 2 and (**G**) the normalized average green light intensity (*GLI*_*N, ave*_) during the illumination.

Based on the theoretical modeling demonstrated in the previous section, a comparison between the electrical output generation behavior under FIn-DEG and WIn-DEG is shown in Fig. 3. First of all, the average of the current output ($\:{I}_{O,ave}$) between the FIn-DEG and WIn-DEG is shown in Fig. 3A-(i), which shows $\:{I}_{O,ave}$ increment from 7.07 ± 1.62 µA to 32.13 ± 3.00 µA as the intermediate layer is changed from FEP to DI water, corresponding to a 4.5-fold enhancement in $\:{I}_{O,ave}$. To further emphasize the significance of this improvement, Table S1 summarizes a comparison with previously reported DEGs employing intermediate layers^28,29,43^. According to this comparison, the WIn-DEG appears to exhibit a relatively high output amplification while maintaining low fabrication complexity and material/process cost, owing to the spontaneous formation of the WIn layer by water droplet impingement during DEG operation. Furthermore, to verify the electrical output generation, the average of the voltage output ($\:{V}_{O,ave}$) of the FIn-DEG and WIn-DEG are shown in Figure S3, which shows $\:{V}_{O,ave}$ increasing from 21.21 ± 1.47 V to 139.72 ± 6.32 V. Considering the voltage output and current output are considered as significant indices for evaluating the electrical output characteristic of the DEG, these experimental results directly infer the advantageous aspect of the high-$\:k$ layer in the DEG operation, which can be assumed in the previous section. Furthermore, a representative current output peak is selected based on its amplitude for deeper inspection of the electrical output generation behavior, as shown in Fig. 3A-(ii). The comparison reveals significant differences not only in the amplitude of the current output but also in the charge transfer duration ($\:\tau\:$), which is defined as the time difference between the surging point ($\:{t}_{s}$) and dissipation point ($\:{t}_{d}$) of the current peak, and the amount of transferred charge ($\:{Q}_{t}$) during $\:\tau\:$, which is calculated as follows:

3 $$\:{Q}_{t}={\int\:}_{{t}_{s}}^{{t}_{d}}{I}_{O}dt$$

In Fig. 3B, the $\:{Q}_{t}$ values during the FIn-DEG and WIn-DEG operations are compared, showing that approximately 0.44 ± 0.05 nC of charge is transferred during the FIn-DEG operation, while 16.08 ± 1.05 nC of charge is transferred during the WIn-DEG operation. For its verification, the 1 µF capacitor is charged with the FIn-DEG and WIn-DEG, and their charging behavior is compared in Fig. 3C. For 100 s, 1.33 µC of the charge can be charged into the capacitor with FIn-DEG, while 11.59 µC of the charge can be charged with WIn-DEG. These experimental results demonstrate a significantly higher amplification of $\:{Q}_{t}$ when compared to the amplification of the $\:{I}_{O,ave}$, which can be affiliated with the extension of the $\:\tau\:$ based on the definition of the current.

In this context, the $\:\tau\:$ of the FIn-DEG and WIn-DEG are shown in Fig. 3D, which shows that approximately 0.15 ± 0.02 ms and 1.98 ± 0.04 ms are required for the charges to be perfectly transferred, respectively. To further demonstrate the effectiveness of WIn layer application, $\:\tau\:$ values demonstrated from previous studies are suggested in Table S2^32,49,55–57^. As the $\:\tau\:$ of the current peak is typically shorter than 1 ms under similar experimental condition, the WIn layer application can be considered as prominent strategy for extending $\:\tau\:$, and there is possibility of further $\:\tau\:$ extension by applying previous reported strategies to the WIn-DEG. Considering that this huge difference in $\:\tau\:$ values may have resulted from the difference in the $\:{C}_{D}$ values, as shown in Eq. 2, the $\:{C}_{D}$ of the FIn-DEG and WIn-DEG are shown in Fig. 3E. The $\:{C}_{D}$ of the FIn-DEG is 4.15 pF at a frequency of 1000 Hz and finally decreases to 4.03 pF at a frequency of 100,000 Hz, while the $\:{C}_{D}$ of the WIn-DEG is decreased from 101.55 pF to 64.83 pF. Notably, a decreasing trend in $\:{C}_{D}$ is observed in the WIn-DEG with increasing frequency, which is distinct from the behavior observed in the FIn-DEG. This phenomenon may be attributed to the oxygen domain-related dielectric relaxation, which has been reported in previous studies^58,59^ as an intrinsic material characteristic of the water. However, the decreasing trend in $\:{C}_{D}$ deviates from the theoretical dielectric constant model of water proposed in previous studies^58^, likely because $\:{C}_{D}$ consists of multiple laminated dielectric layers—a structural configuration known to modify the dielectric behavior across the frequency^60^. Although $\:{C}_{D}$ trend across a wide range of frequencies, a clear correlation with the $\:\tau\:$ extension is still observed. For the further verification of the effectiveness of the WIn layer application, the WIn-DEG is compared with a thin FIn-DEG possessing a thinner FEP dielectric contact layer, which is representative of the conventional DEG structure, as shown in Figure S4. Although the thin FIn-DEG has a smaller dielectric contact layer thickness than the WIn-DEG, it exhibits a lower $\:{C}_{D}$, resulting in reduced $\:{I}_{O,ave}$, and $\:{Q}_{t}$, and a shorter $\:\tau\:$. These experimental results clearly demonstrate that introducing the WIn layer is an effective strategy for enhancing the electrical output of DEGs. To directly show its effectiveness, a green light-emitting diode is illuminated with the FIn-DEG and WIn-DEG to visually demonstrate the $\:\tau\:$ extension by comparing the illuminating time of the LED as shown in Fig. 3F and Supporting Video 2. To prevent undesired effects from other components, the DEG and green LED re connected directly without any auxiliary circuit components. Furthermore, the green LED illumination is captured with a high-speed camera at 960 fps and a playback of $\:\times\:$0.05 to show their distinct differences. For a detailed analysis, the average green light intensity of each image in Fig. 3F is normalized, as shown in Fig. 3G. Based on Fig. 3F and G, it is well shown that the LED illuminated with WIn-DEG can be illuminated six times longer than that illuminated with FIn-DEG, which can directly show that the $\:\tau\:$ extension has an effective aspect even in the actual condition. However, the LED illumination duration is dramatically shorter than the $\:\tau\:$ of both FIn-DEG and WIn-DEG when comparing Fig. 3D and G, which can be attributed to the threshold voltage of the LED. In this context, further studies on the intermediate layer that possesses higher $\:k$ values can demonstrate the possibility of further enhancement on the $\:\tau\:$ to the practically utilizable level.

However, although the high-$\:k$ of the DI water, still there are possibility of the charge leakage through the WIn layer, due to its conductivity. To verify this, leakage current density under the electric field on the range from 1900 V/m to 1960 V/m is measured across the bottom electrode, Kapton layer, intermediate layer, FEP layer, and top electrode, as shown in Figure S5. This result shows a slight increase in leakage current density under the WIn-DEG compared to the FIn-DEG. However, both devices exhibit leakage current densities on the order of pA/cm^2^, which can be exhibited that similar amount of leakage current density is occurred under the FIn-DEG and WIn-DEG, although the slight conductivity of the WIn layer. As previous study mentions a leakage current density below 10^− 7^ A/cm^2^ is typically regarded as an insulating condition^61^, these results further confirm that the WIn-DEG design effectively eliminates the conductivity effect of the DI water to the WIn-DEG operation, which could otherwise act as an unexpected parasitic factor.

Furthermore, to ensure the reliability of the experimental data demonstrated for verifying the theoretical modeling, the device-to-device variation of the WIn-DEG is further demonstrated, as shown in Figure S6. A total of four devices are independently fabricated (labeled as Sample 1 to Sample 4, S1-S4), using an identical fabrication process, and the $\:{C}_{D}$ behavior and electrical outputs of each device are analyzed. Despite their individuality, almost identical $\:{C}_{D}$ trends are observed across S1-S4 over the frequency range of 1,000 to 100,000 Hz, and all samples exhibited $\:{C}_{D}$ values of approximately 103 pF at a frequency of 1,000 Hz. Following this $\:{C}_{D}$ trend, almost identical $\:{I}_{O,ave}$, $\:{Q}_{t}$, and $\:\tau\:$ values are observed across S1-S4, thereby demonstrating excellent reproducibility of the results and reliability of the theoretical model.

Also, to show the long-term stability of the WIn-DEG across 4 h of repeated test cycles, the $\:{C}_{D}$ and the electrical output is shown in Figure S7. As a result, nearly identical $\:{C}_{D}$ trends are observed over the 1,000 to 100,000 Hz frequency range at every hour of measurement, with each sample showing a $\:{C}_{D}$ value of approximately 103 pF in the frequency of 1,000 Hz. With this $\:{C}_{D}$ trend, almost similar $\:{I}_{O,ave}$, $\:{Q}_{t}$, and $\:\tau\:$ values are observed at every hour, indicating the excellent stability of the WIn-DEG, which further enhances the reliability of the experimental result.

Furthermore, for a deeper inspection of the relationship between the intermediate layer and $\:{Q}_{t}$ and $\:\tau\:$, the thickness of both intermediate layers ($\:{t}_{In}$) is reduced from 5 mm to 1 mm in Figure S8. Both the $\:{Q}_{t}$ and $\:\tau\:$ of the FIn-DEG are distinctly increased with the thickness decrement as the $\:{C}_{D}$ increases, which corresponds well with the theoretical model based on the definition of the capacitance. However, the $\:{Q}_{t}$ and $\:\tau\:$ of the WIn-DEG slightly decrease, which differs from the behavior exhibited by that of FIn-DEG, and its decreasing range is relatively small when compared to the increasing range of the FIn DEG. In particular, this parameter also follows the decreasing trend exhibited by the $\:{C}_{D}$ of WIn-DEG with decreasing thickness—in contrast to the theoretical modeling—possibly due to the dipolar composition of the water. According to previous study^58^, the water layer behaves as a dipolar medium composed hydrogen- and oxygen-dominated domains, leading to polarization suppression in thin WIn layers because the domain structures cannot fully develop under strong interfacial constraints such as resonance damping and coupling between adjacent domains. However, as the WIn layer becomes thicker, these interfacial constraints are relaxed, allowing the dipolar domains to align more freely and thereby enhancing the overall polarization. Consequently, an increase in $\:{t}_{In}$ of the WIn-DEG exhibits a positive correlation with $\:{C}_{D}$.

Moreover, the potential impurity effect of the WIn layer on the WIn-DEG electrical output is further verified by introducing ionic components, and applying a soil suspension to the WIn layer, as shown in Figures S9 and S10. To investigate the influence of the ionic content, the WIn layer is replaced to KCl aqueous solutions of 1 M and 2 M concentrations. As previously reported, the ionic concentration of an aqueous solution exhibits a negative correlation with its dielectric constant, which is attributed to restricted molecular rotation of H_2_O molecules coordinated with cation components thus remain unresponsive to the external electric field^62,63^, indicating a decrease in the capacitance of the WIn layer with increasing KCl concentration. Correspondingly, the $\:{I}_{O,ave}$, $\:{Q}_{t}$, and $\:\tau\:$ values show decreasing trends—from 26 µA to 19.73 µA, from 14.93 nC to 8.4 nC, and from 1.85 ms to 1.18 ms, respectively. As the excessive conductive increasing resulted from this WIn layer substitution can arise unexpected effect, the leakage current behavior of the WIn layer composed of DI water (conductivity = 6.17 µS/cm), 1 M KCl aqueous solution (conductivity = 71.15 mS/cm), and 2 M KCl aqueous solution (conductivity = 133.02 mS/cm) are monitored as shown in Figure S9D. Despite this increase in conductivity, the leakage current densities under all conditions remained on the order of pA/cm^2^, further confirming that the electrical output degradation resulted from the ion composition is predominantly governed by the $\:{C}_{D}$ behavior rather than by its conductivity. In addition, to further verify the impurity effect that can occur under realistic environmental conditions, a soil suspension is introduced into the WIn layer as shown in Figure S10. With the incorporation of soil impurities, the $\:{C}_{D}$ at the 1000 Hz is decreased from 103.12 pF to 93.82 pF, leading to degradation in $\:{I}_{O,ave}$, $\:{Q}_{t}$, and $\:\tau\:$—from 26 µA to 20.8 µA, from 14.93 to 12.29 nC, and 1.85 to 1.63 ms. These results further verify the theoretical modeling presented in Sect. 2.2 and emphasize the necessity of future studies aimed at mitigating contamination-induced degradation in the WIn-DEG.

### Relationship between the droplet impact conditions and electrical output

**Fig. 4:**
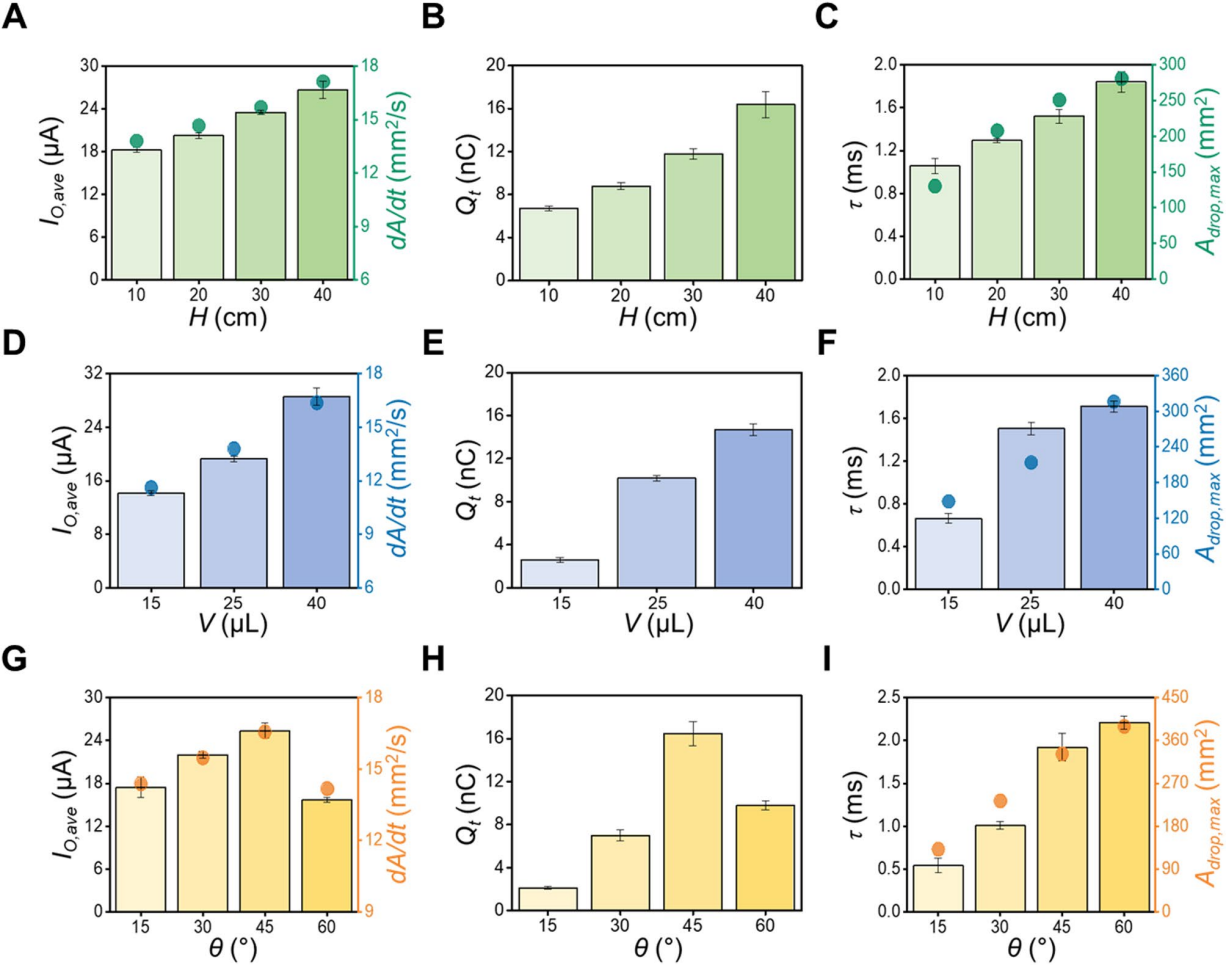
(**A**) $\:{I}_{O,ave}$, (**B**) $\:{Q}_{t}$, and (**C**) $\:\tau\:$ under the operation of WIn-DEG under droplet impact with various heights. (**D**) $\:{I}_{O,ave}$, (**E**) $\:{Q}_{t}$, and (**F**) $\:\tau\:$ under the operation of WIn-DEG with various droplet volumes. (**G**) $\:{I}_{O,ave}$, (**H**) $\:{Q}_{t}$, and (**I**) $\:\tau\:$ under the operation of WIn-DEG under droplet impact with various droplet incident angles with the WIn-DEG surface. Dot graph in (**A**), (**D**), (**G**) represents $\:dA/dt$ value during DEG operation, and (**C**), (**F**), (**I**) represents $\:{A}_{drop,max}$ value.

The electrical properties of the DEG components and the hydraulic behavior of the droplets upon impact, such as the maximum droplet contact area ($\:{A}_{drop,max}$) and rate of contact area change ($\:dA/dt$), are the key factors that determine the electrical output generation of the DEG^64^. Therefore, the effects of these impact-related parameters on the electrical output generation behavior of WIn-DEG must be investigated. The $\:{I}_{o,ave}$, $\:{Q}_{t}$, and $\:\tau\:$ variation under various impact conditions, such as the dripping height ($\:H$), droplet volume ($\:V$), and droplet incident angle ($\:\theta\:$), are shown in Fig. 4. Figure 4A shows the correlation between the $\:H$ of the droplet and the current output. As the $\:H$ increases from 10 cm to 40 cm, the $\:{I}_{o,ave}$ correspondingly increases from 18.24 ± 0.36 µA to 26.64 ± 1.19 µA, respectively. This can result from the increased contact pressure and droplet spreading speed when a droplet impinged from a higher $\:H$, resulting in increased $\:dA/dt$ from 13.81 mm^2^/s to 17.14 mm^2^/s and consequently to an improved current output^64^. Owing to similar reasons, the upward trend is observed as $\:{Q}_{t}$ increases from 6.70 ± 0.02 to 16.38 ± 1.21 nC with the $\:H$ increment from 10 cm to 40 cm, as shown in Fig. 4B. As shown in Fig. 4C, $\:\tau\:$ also increases from 1.06 ± 0.07 ms to 1.84 ± 0.09 ms with a rise in $\:H$ from 10 cm to 40 cm. As $\:\tau\:$ is primarily depends on the $\:{C}_{D}$, and $\:{C}_{D}$ is proportionally related to the maximum contact area of the droplet^65^ according to the theoretic capacitor model as follows:

4 $$\:{C}_{D}\approx\:{C}_{In}={k}_{In}\frac{{A}_{drop,max}}{{t}_{In}}$$

where $\:{k}_{In}$ and $\:{A}_{drop,max}$ represent the dielectric constant of the intermediate layer and maximum droplet contact area, respectively. The extension of $\:\tau\:$ can be observed as the maximum spreading area of the droplet increases from 130.45 mm^2^ to 280.71 mm^2^ with increasing $\:H$, which results in an augmented current output.

Furthermore, $\:V$ is changed from 15 µL to 40 µL by carefully adjusting the diameter of the outlet. In alignment with the previous observation, $\:{I}_{o,ave}$, $\:{Q}_{t}$, and $\:\tau\:\:$are enhanced from 14.16 ± 0.36 µA to 28.56 ± 1.28 µA, from 2.61 ± 0.22 nC to 14.70 ± 0.54 nC, and from 0.66 ± 0.46 ms to 1.71 ± 0.05 ms, respectively, as shown in Fig. 4D and F. This behavior can be driven by the $\:dA/dt$ increment from 11.62 mm^2^/s to 16.37 mm^2^/s and the $\:{A}_{drop,max}$ increment from 148.19 mm^2^ to 315.98 mm^2^, thereby increasing the overall output performance of the system^65^.

In addition, the electrical output from the WIn-DEG is measured from a $\:\theta\:$ of 15° to 60° to evaluate the effect of $\:\theta\:$. As shown in Fig. 4G, $\:{I}_{o,ave}$ increases from 17.44 ± 1.43 µA to 25.36 ± 1.08 µA as the $\:\theta\:$ increases from 15° to 45°. In contrast, further increasing $\:\theta\:$ to 60° causes $\:{I}_{o,ave}$ to decrease to 15.68 ± 0.33 µA, which can be attributed to the combined effects of the increasing trend in the maximum droplet contact area and decreasing droplets spreading speed^66^. This leads to an largest $\:dA/dt$ in 45° of 16.56 mm^2^/s, which is consistent behavior with previous studies^26,64^. In addition, the $\:{Q}_{t}$ has similar behavior with the $\:{I}_{o,ave}$ under various $\:\theta\:$, which exhibits a maximum value of 16.47 ± 1.14 nC at an optimal angle of incidence of 45°, as shown in Fig. 4H. However, aside from the $\:{I}_{o,ave}$ and $\:{Q}_{t}$ behavior, $\:\tau\:$ exhibits an increasing trend from 0.54 ± 0.09 ms to 2.20 ± 0.08 ms, which can be attributed to the increasing trend in the $\:{A}_{drop,max}$ from 131.64 mm^2^ to 389.55 mm^2^ with the increment of the $\:\theta\:$, in other words, decreasing the angle between the WIn-DEG and ground, as shown in Fig. 4I. With these experimental results, the optimal electrical output can be generated under the $\:H$ of 40 cm, $\:V$ of 40 µL, and $\:\theta\:$ of 45°.

To further show the effect of the environmental parameter of WIn-DEG operation to its electrical output, current output under the enhanced temperature and humidity are monitored, as shown in Figure S11. As the relative humidity of the operational environment increases from 40% to 94% by applying humid chamber, a slight decrease is observed in the current output, amount of transferred charge, and charge transfer duration—from 25.2 µA to 22.13 µA, from 15.62 nC to 13.33 nC, from 1.85 ms to 1.75 ms, respectively—which can be attributed to the enhanced surface charge dissipation under humid conditions^67^. However, owing to the inherent hydrophobicity and chemical stability of FEP, the extent of this decrease remains limited. Furthermore, to monitor the temperature effect on the current output behavior of WIn-DEG, the WIn layer is heated to 40 °C. According to previous studies, an increase in the temperature of the water layer slightly enhances its dielectric constant^59,68^. Consequently, a slight increasing is observed in the current output, the amount of transferred charge, and the charge transfer duration—from 25.2 µA to 26.27 µA, from 15.62 nC to 17.27, and from 1.85 ms to 2 ms, respectively—as the WIn layer temperature increases from 21 °C to 40 °C. Although the environmental parameters are widely altered under conditions comparable to real-world operating conditions, the electrical output of the WIn-DEG exhibited only minor fluctuations, indicating its strong environmental stability.

### Necessity of charge transfer process tailoring on DEG practical application

**Fig. 5:**
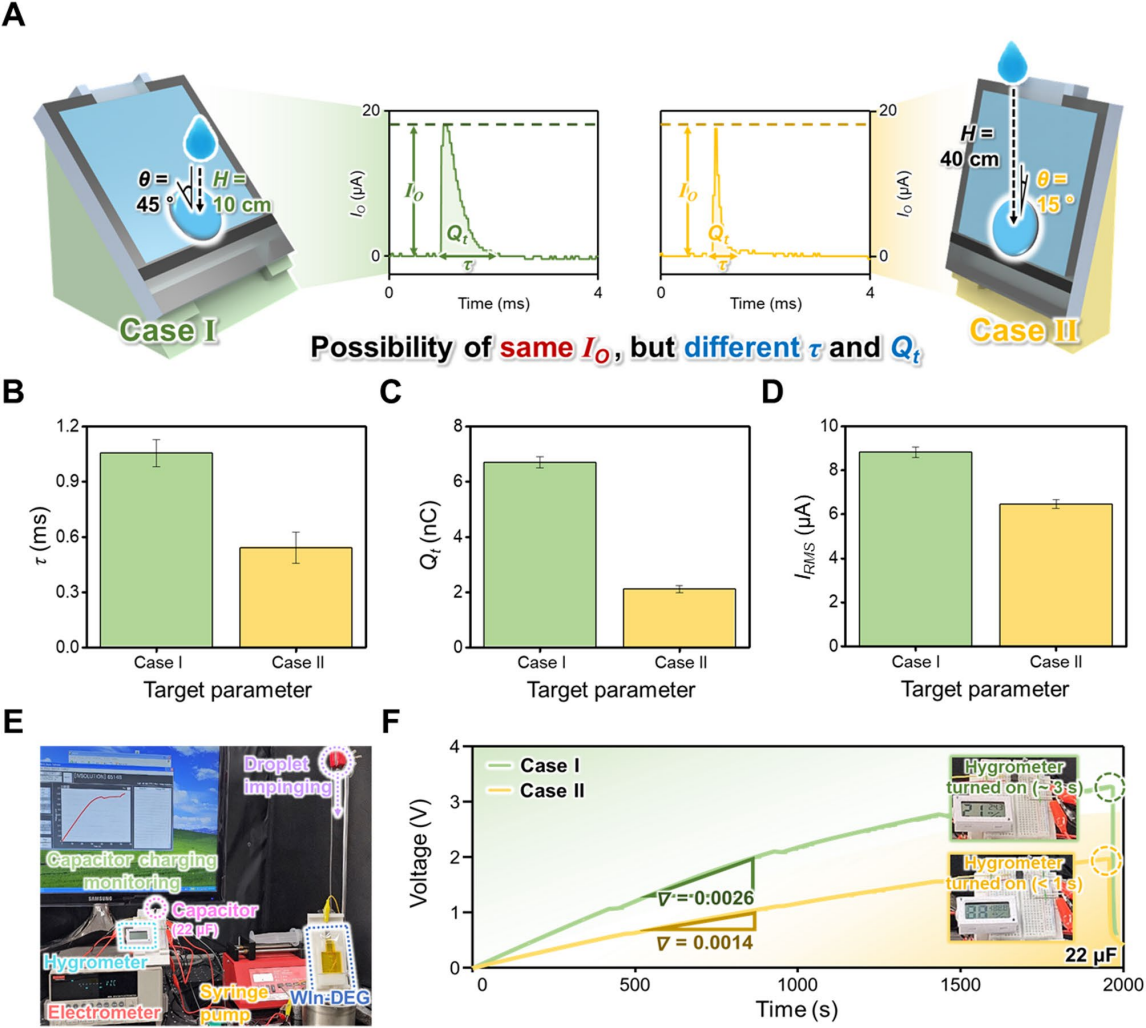
(**A**) Schematic of the two cases, which possess similar $\:{I}_{O}$ and different $\:\tau\:$ and $\:{Q}_{t}$ values from Fig. 4. (**B**) $\:\tau\:$, (**C**) $\:{Q}_{t}$, and (**D**) root mean square value $\:{I}_{O}$ ($\:{I}_{RMS}$) from two cases. (**E**) Setup used to observe the turning-on behavior of the hygrometer under Cases I and II, and (**F**) their capacitor charging behavior and hygrometer operation behavior.

In Sect. 2.4, it is well demonstrated that the current output, amount of transferred charge, and charge transfer duration are affected by the parameters related to droplet impact. Interestingly, based on the experimental results, some cases generate the same current output but have different amounts of transferred charge and charge transfer duration, such as $\:H$ = 10 cm and $\:\theta\:$ = 15°. For the intuitive comparison, the $\:H$ = 10 cm case is denoted as Case I, and the $\:\theta\:$ = 15° case is denoted as Case II, as shown in Fig. 5A, and their $\:{I}_{O,ave}$, $\:{Q}_{t}$, $\:\tau\:$, and RMS values of the $\:{I}_{O,ave}$ ($\:{I}_{RMS}$) are shown in Figures S12, 5B to 5D for the detailed comparison. As shown in Figure S12, there are no significant differences in the $\:{I}_{O,ave}$ of Cases I and II, which are 18.24 ± 0.36 µA and 17.44 ± 1.43 µA, respectively. However, these two cases possess a distinct difference in $\:\tau\:$ with values of 1.06 ± 0.07 ms and 0.54 ± 0.09 ms, respectively, as shown in Fig. 5B, which can be attributed to the variations in droplet dynamics during its impact. Considering this $\:\tau\:$ behavior, $\:{Q}_{t}$ of 6.70 ± 0.20 nC and 2.12 ± 0.13 nC is transferred under Cases I and II, respectively, as shown in Fig. 5C. Considering that this distinct electrical output generation behavior may affect the actual amount of harvested energy, Fig. 5D shows the $\:{I}_{RMS}$ values of Cases I and II, which are calculated using the following Eq. 5^26,30,69,70^:

5 $$\:{I}_{RMS}=\sqrt{\frac{1}{\tau\:}{\int\:}_{{t}_{s}}^{{t}_{d}}{{I}_{O}\left(t\right)}^{2}dt}$$

Interestingly, although the $\:{I}_{O,ave}$ of Cases I and II are similar, Case I exhibits a higher $\:{I}_{RMS}$, which is 8.82 ± 0.23 µA, while Case II exhibits an $\:{I}_{RMS}$ of 6.46 ± 0.19 µA. In this regard, this difference in $\:{I}_{RMS}$ is attributed to the difference in the charge transfer process. Considering that the $\:{I}_{RMS}$ is one of the significant indices for evaluating the actual energy harvesting capacity of the energy harvester, it can be demonstrated that a larger amount of energy can be harvested with a single droplet under Case I. Based on these comparisons, it can be well deduced that the augmentation of the current output must be focused by considering amplitude of the electrical output, $\:\tau\:$, and $\:{Q}_{t}$ to enhance the actual energy harvesting capacity. To demonstrate the practical effectiveness of this behavior, the capacitor charging behaviors in Cases I and II and the possibility of their utilization as power sources for electronic devices are investigated, as shown in Fig. 5E and F. For its examination, the WIn-DEG is connected to the capacitor circuit, which comprises a 22 µF capacitor and rectifier, and the capacitor is connected to the electrometer for real-time charging behavior monitoring. For the electronic device, a hygrometer s prepared and ready to be connected to the capacitor, as shown in Figure S13. As shown in Fig. 5F, the capacitor can be charged faster under Case I; thus, it can be charged up to 3.26 V under Case I, and charged up to 1.97 V under Case II for 1950 s. After 1950 s of capacitor charging, the hygrometer is connected to the capacitor. Considering this distinct charging behavior, the hygrometer can be turned on for approximately 3 s with the capacitor charged in Case I, whereas the display of the hygrometer blinked within 1 s with the capacitor charged in Case II, as shown in Supporting Video 3. These experimental findings effectively demonstrate the effectiveness of enhancing $\:\tau\:$ and $\:{Q}_{t}$ for energy harvesting, which indirectly asserts the superiority of the water intermediate layer application method for droplet energy harvesting.

**Fig. 6:**
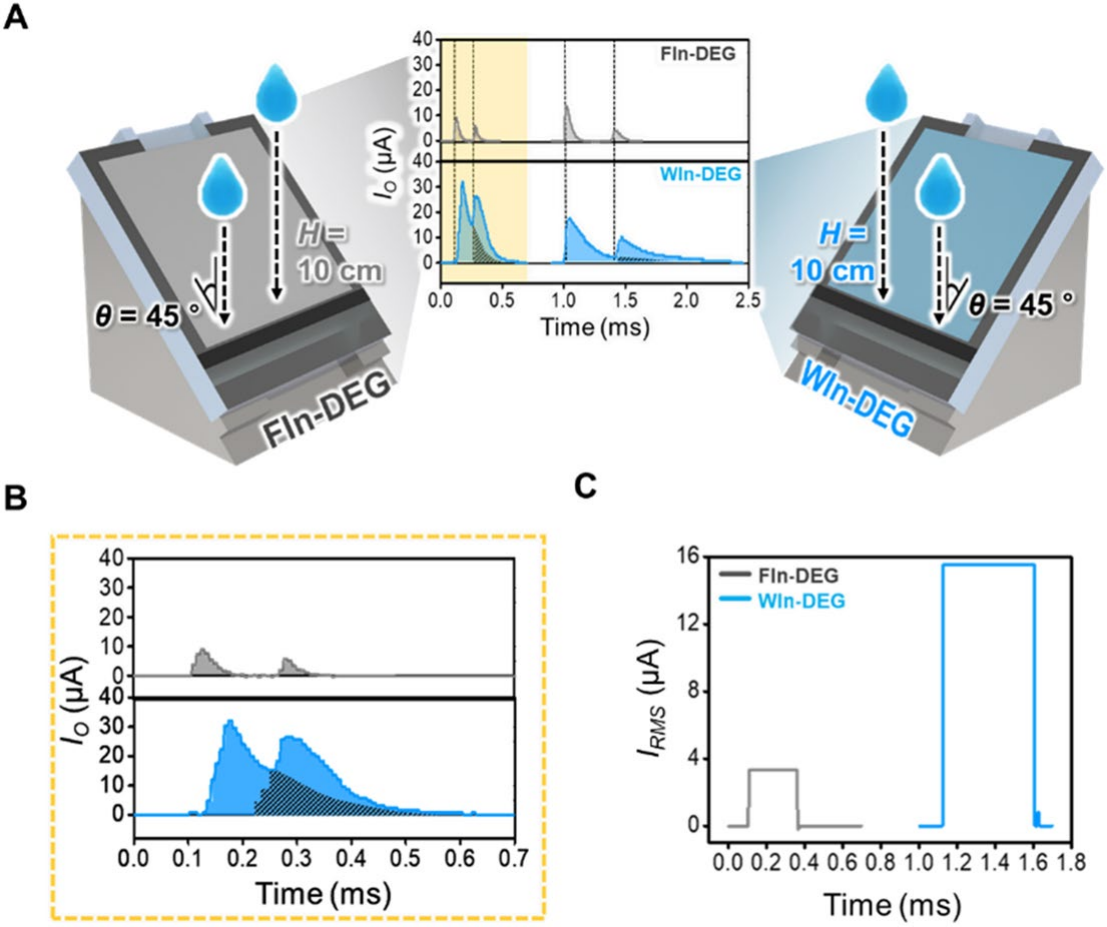
(**A**) Schematic of the FIn-DEG and WIn-DEG under enhanced droplet input frequency for the demonstration of peak overlapping. (**B**) Detailed demonstration of the overlapped peak. Overlapping areas are shown as hatched lines. (**C**) The $\:{I}_{RMS}$ of the FIn-DEG and WIn-DEG based on the current output previously shown.

Moreover, considering the $\:\tau\:$ extension on WIn-DEG, the interval between the peaks can be decreased, which can potentially increase the probability of the peak overlap if the frequency of the droplet input is increased. To show the peak overlap, two droplets are impinged within a short time interval onto both WIn-DEG and FIn-DEG using an $\:H$ of 10 cm and a $\:\theta\:$ of 45°, as shown in Fig. 6A. As the $\:\tau\:$ of the WIn-DEG is longer than that of the FIn-DEG, the overlap of the two peaks can be sporadically observed during the operation of WIn-DEG but cannot be observed during the FIn-DEG operation under the same droplet input condition. A representative peak is shown in Fig. 6B for its detailed demonstration. Unlike the FIn-DEG operation, two peaks can be overlapped for 0.3 ms without an interval under the operation of WIn-DEG, and the overlapped part is shown with an oblique line in the graph. To demonstrate the practical effectiveness of this electrical output generation behavior, the $\:{I}_{RMS}$, which is calculated from the surging point of the first peak to the dissipation point of the second peak, is shown in Fig. 6C. Approximately 3.32 µA of $\:{I}_{RMS}$ is generated during the FIn-DEG operation, while 15.55 µA of $\:{I}_{RMS}$ is generated during the WIn-DEG operation. Considering that the peak of the $\:{I}_{O}$ under the FIn-DEG and WIn-DEG operations are 9.00 µA and 32.00 µA respectively, the normalized values of $\:{I}_{RMS}$ are 0.37 and 0.49, which indicates that the peak overlap observed in Fig. 6B results in a 32.43% efficient energy harvesting. Given that the continuous peak overlap can lead to the alternation of the AC-type electrical output of the DEG to the DC-type electrical output, and it can increase the total available energy for practical utilization as a power source, the utilization of the WIn layer for augmentation of the electrical output by the $\:\tau\:$ extension and enhanced $\:{Q}_{t}$ can be a promising strategy for the practical utilization of the DEG as a power source.

## Conclusion

Owing to the structural analogy between the FET and the DEG, utilizing the high-$\:k$ intermediate layer on the DEG can be an effective methodology for electrical output augmentation by simultaneously increasing the amount of transferred charge and extending the charge transfer duration at the same time, which may overcome the limitations of the DEG. For the high-$\:k$ intermediate layer, utilizing water, which is one of the necessary components for DEG operation, allows spontaneous high-$\:k$ intermediate layer generation without a complex fabrication process. Considering that the difference in the charge transfer behavior can affect the RMS value of the current output, which is strongly related to effective energy harvesting, the increment in the amount of transferred charge and the extension of the charge transfer duration resulting from the high-$\:k$ water intermediate layer can possibly increase the electrical energy harvesting capacity of the DEG. In addition, the extension of the charge transfer duration can sporadically lead to peak overlapping, suggesting the potential for DC electrical output generation in DEG operations. Building on this advantageous aspect of high- $\:k$ water intermediate layer, further systematic design research on the WIn-DEG aimed at prolonging the retention of the WIn layer potentially enable additional improvement in system stability, reducing the need for frequent replenishment and enhancing long-term maintenance efficiency. Ultimately, continued investigation into charge transfer duration extension will pave the way for DEG studies, contributing to the development of energy harvesters with enhanced energy harvesting capacity.

## Materials and methods

### Sample preparation

For the DEG, FEP (Alphaflon, thickness: 125 μm) film was utilized as a dielectric contact layer, Polyimide Kapton tape (Alphaflon, thickness: 65 μm) was utilized as an insulation layer, and polylactic acid (PLA-i21 filament, Cubicon) was 3D printed and utilized as a substrate. The FEP film was attached to the PLA substrate with polydimethylsiloxane (PDMS, Sylgard 184), which was prepared by mixing the base and curing agent at a ratio of 10:1. The intermediate layer of the FIn-DEG was fabricated by hot pressing 40 sheets of the FEP film at 270 °C and an input force of 3000 N to fabricate an FEP block with a thickness of 5 mm. For the intermediate layer of the WIn-DEG, a 5 mm layer of DI water (KCU-PW3, Korea Cleanup Chem, conductivity: 2.6 µS/cm) was applied.

### Sample characterization

The capacitance of the dielectric contact layer was measured using an LCR meter (E4980A, Keysight) equipped with a probe station (MST4000A, MS-TECH) while the two probes were connected to the electrodes, which are attached under the Kapton insulating layer and above the FEP dielectric contact layer.

### Electrical output performance characterization

A deionized (DI) water droplet was applied to the input droplet under controlled input frequency conditions using a syringe pump (NE-4000, New Era Pump Systems). The droplet volume was adjusted by employing syringe tips with different inner diameters, which were attached to a hose connected to syringe. The droplet impingement height was controlled by fixing the syringe tip to experimental stand at a specific height. With this setup, the parameters that can affect the electrical output performance of the WIn-DEG were precisely controlled. The resultant current output generated from the droplet impingement on the DEG was measured using an oscilloscope (DS1074z, Rigol, Internal resistance = 1 MΩ) while the low-noise current preamplifier (SR570, Standard Research Systems) was connected. The resultant voltage output generated from the droplet impingement on the DEG was measured using an oscilloscope while the high voltage probe (DP-50, Pintek, Internal resistance = 15 MΩ) was connected. The leakage current density of the system is measured with the electrochemical station (CHI660E, CH Instruments Inc.), under scan rate of 0.01 V/s, sample interval of 0.001 V, the quiet time of 5 s, and the sensitivity (A/V) of 10^− 9^. To measure the capacitor charging behavior, an electrometer (6514, Keithley Instruments) was connected to the capacitor. All electrical output performance characterization experiments are conducted under the room condition with room temperature = 21 °C, and relative humidity = 40%.

### High-speed camera analysis of the LED illumination

To compare the LED illumination behavior, the green LED was directly connected to the DEG and filmed in the super slow-motion mode of the Samsung Galaxy 22+, which films videos at 960 fps. For a detailed comparison, the video was played back at $\:\times\:$0.05, using video editing software. In addition, the average green light intensity of the illuminated LED was analyzed using image processing and analysis software.

### High-speed camera analysis of the droplet dynamics

To demonstrate the maximum droplet spreading and its rate of change for the parametric study, the droplet dynamics during the DEG operation is filmed with the high-speed camera (Chronos 2.1-HD High-speed camera, Chronos) under a frame per second of 2531.65, and the droplet contact area under each parameter was calculated with the imaging analysis software, Image J.

## Supplementary Information

Below is the link to the electronic supplementary material.

Supplementary Material 1

Supplementary Material 2

Supplementary Material 3

Supplementary Material 4

## Data Availability

The datasets used and/or analysed during the current study available from the corresponding author on reasonable request.
