# Supplementary material for: High-k water intermediate layer-mediated charge transfer modulation toward stable and augmented output generation of droplet-based electricity generators
Source: Sci Rep. 2025 Dec 30;15:45695. doi: 10.1038/s41598-025-27319-9 (PMC12753728; doi:10.1038/s41598-025-27319-9)
Supplement: Supplementary file 4 — Supplementary Material 4 [file 41598_2025_27319_MOESM4_ESM.docx]

Supporting Information

**High-*k* Water Intermediate Layer-Mediated Charge Transfer Modulation toward Stable and Augmented Output Generation of Droplet-based Electricity Generators**

Sunmin Jang^1,#^, Soban Ali Shah^1,#^, Dong Yong Park^2,#^, Sumin Cho^1^, Dongik Kam^1^, Girak Gwon^1^, Yoonsang Ra^1,3^, Hee Jae Hwang^4^, Moonwoo La^5,*^, Sung Jea Park^5,6,7,*^, and Dongwhi Choi^1,*^

^1^ Department of Mechanical Engineering (Integrated Engineering Program), Kyung Hee University, 1732 Deogyeong-daero, Yongin, Gyeonggi 17104, Republic of Korea

^2^ Advanced Mobility Components Group, Korea Institute of Industrial Technology, 320 Techno sunhwan-ro, Yuga-eup, Dalsung-gun, Republic of Korea

^3^ School of Mechanical Engineering, Chonnam National University, 77 Yongbong-ro, Buk-gu, Gwangju 61186, Republic of Korea

^4^ School of Mechanical System Engineering, Kumoh National Institute of Technology, 61, Daehak-ro, Gumi-si, Gyeongsangbuk-do 39177, Republic of Korea

^5^ School of Mechanical Engineering, Korea University of Technology and Education, 1600 Chungjeol-ro, Cheonan, Chungnam 31253, Republic of Korea

^6^ Advanced Technology Research Centre, Korea University of Technology and Education, 1600 Chungjeol-ro, Cheonan, Chungnam 31253, Republic of Korea

^7^ Future Convergence Engineering, Korea University of Technology and Education, 1600 Chungjeol-ro, Cheonan, Chungnam 31253, Republic of Korea

^#^ These authors contributed equally to this work.

*To whom all correspondence should be addressed.

Email : [mla@koreatech.ac.kr](mailto:mla@koreatech.ac.kr) *(M. La)*

Email : [psj7517@koreatech.ac.kr](mailto:psj7517@koreatech.ac.kr) *(S. J. Park)*

Email : [dongwhi.choi@khu.ac.kr](mailto:dongwhi.choi@khu.ac.kr) *(D. Choi)*

**
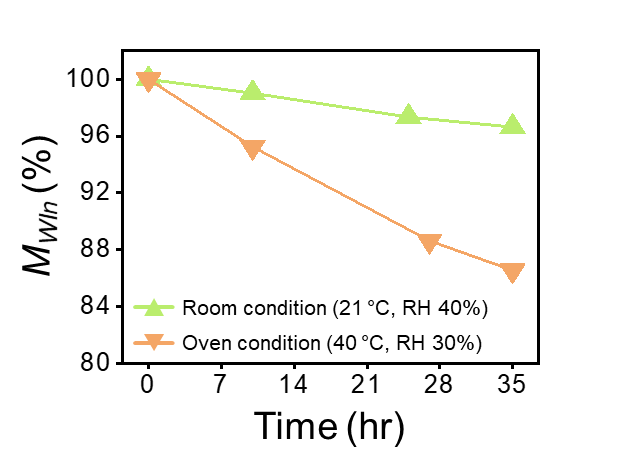
**

**Figure S1.** The WIn layer evaporation behavior under the room condition and oven condition during 35 hours.

**
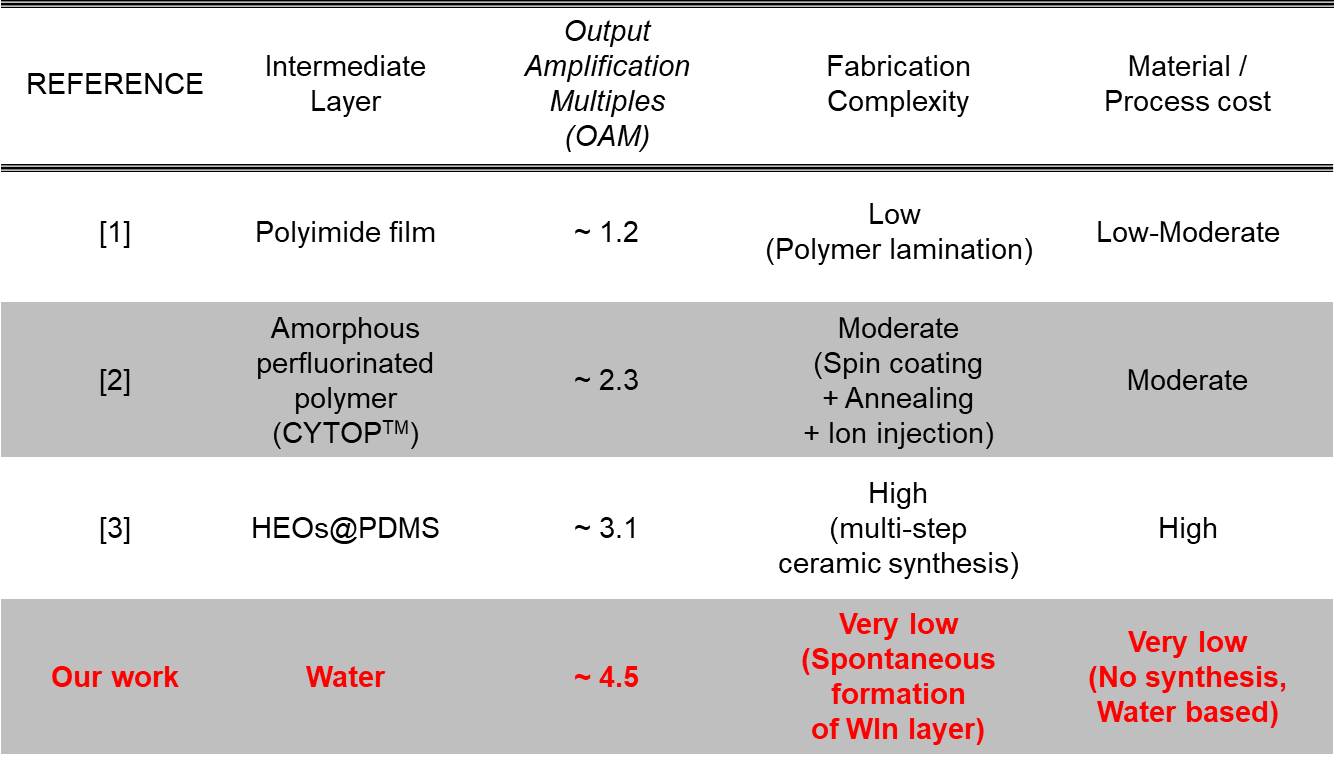
**

**Table S1.** Output amplification, Fabrication complexity, and material/process cost comparison between the WIn-DEG and previously reported intermediate layer applied DEG.

**
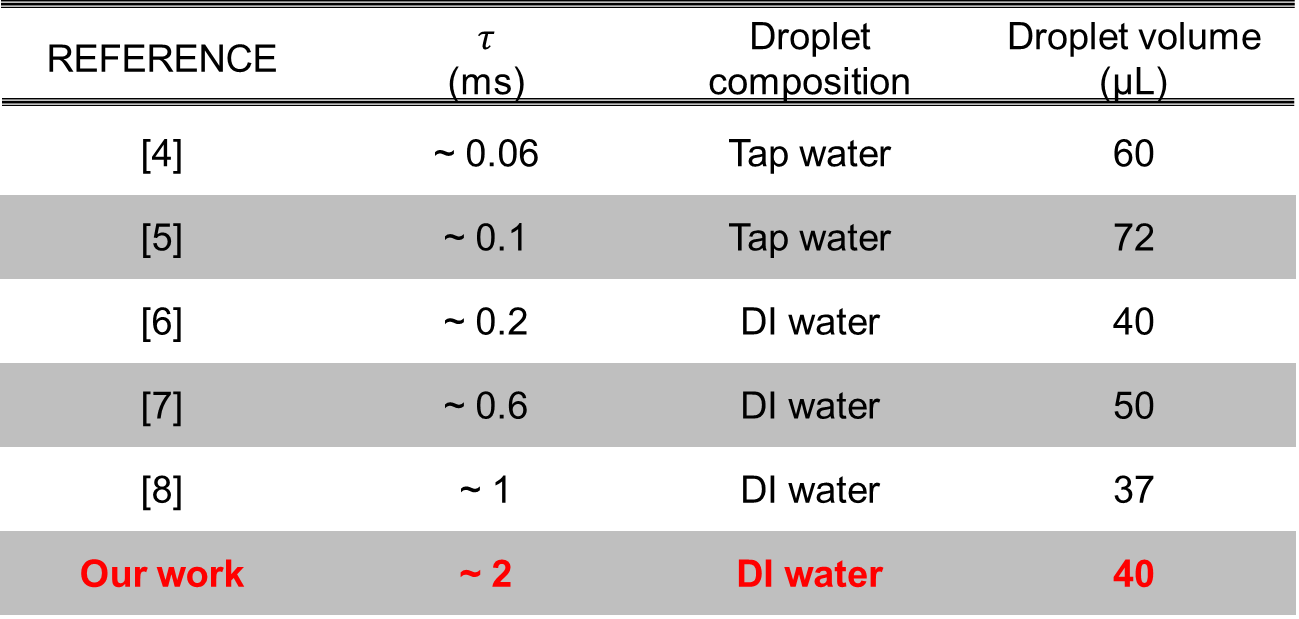
**

**Table S2.** Charge transfer duration comparison between the WIn-DEG and previously reported DEGs and their related parameters.

**Surface electrification of the FEP resulted from liquid-solid contact electrification.**

The negative charging of the FEP surface is resulted from the liquid-solid contact electrification, which is well known phenomenon resulted from the contact and detachment of the water and solid surface. When the liquid droplet contacts the FEP surface, an electric double layer (EDL) forms at the interface via ion adsorption. This conventional EDL model is recognized as the governing mechanism of the liquid-solid contact electrification. However, recent studies have shifted the conventional EDL model to consider both ion adsorption and electron transfer [9]. This revision arises from the large deviation between the theoretical and experimentally observed surface charges in liquid-solid contact electrification, as well as from the possibility of surface electrification occurring between solids and nonpolar liquids. Previous studies have demonstrated that electron transfer from the water droplet to the FEP surface can be explained using the band alignment model, which incorporates electron cloud overlap, as shown in Figure S2. Before contact, the highest occupied molecular orbital (HOMO) of the water, primarily originating from the oxygen orbitals, lies at a higher energy level than the lowest unoccupied molecular orbital (LUMO) of FEP. During their contact, the interface energy overlap facilitates electron hopping from the HOMO of water to the LUMO of FEP, driven by their energy level difference. After their separation, the transferred electrons remain trapped within the FEP under ambient conditions, leading to a negatively charged FEP surface and positively charged water. This surface electrification mechanism accurately represents the condition of the droplet impingement onto the FEP dielectric contact layer. Furthermore, due to the high polarity of the water, the positively charged water molecule polarize the neighboring water molecules, resulting in the overall polarization of the water droplet along its contact interface with the FEP and top electrode.


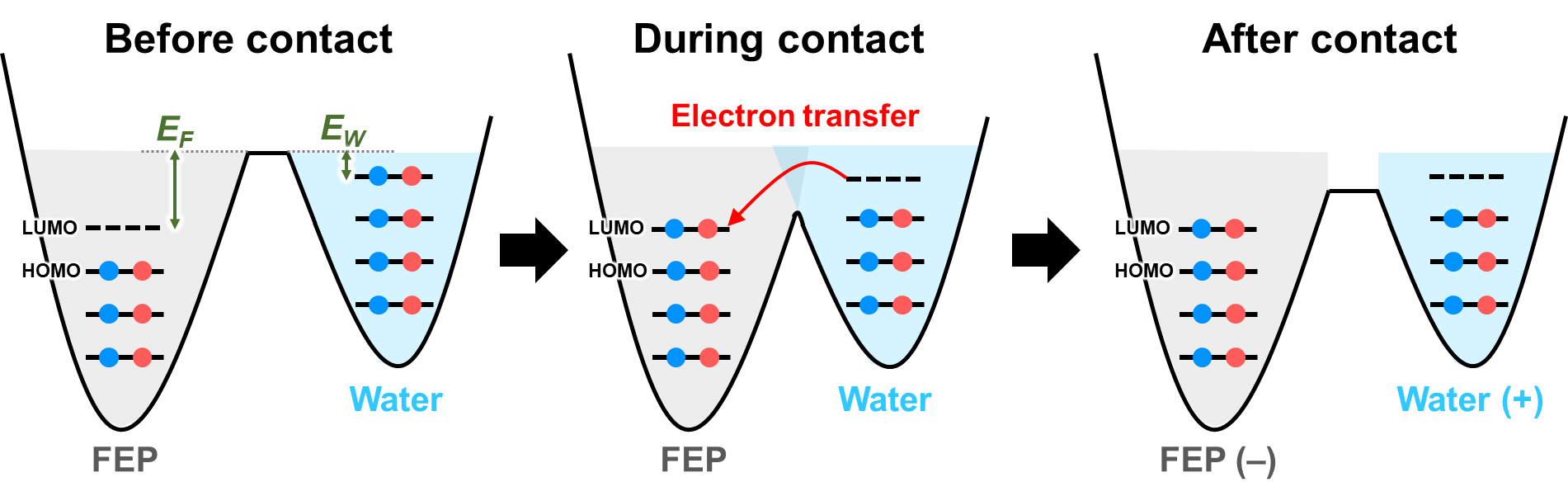


**Figure S2.** The schematic of the electron transfer model on the FEP-water interface. $E_{F}$ and $E_{W}$ correspond to the potential energy levels required for electron emission from the LUMO of FEP and from the oxygen atom of water molecule, respectively


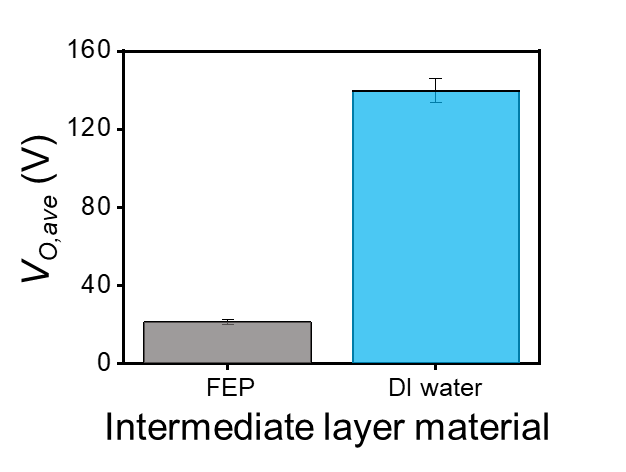


**Figure S3.** The voltage output generated from FIn-DEG and WIn-DEG.

**
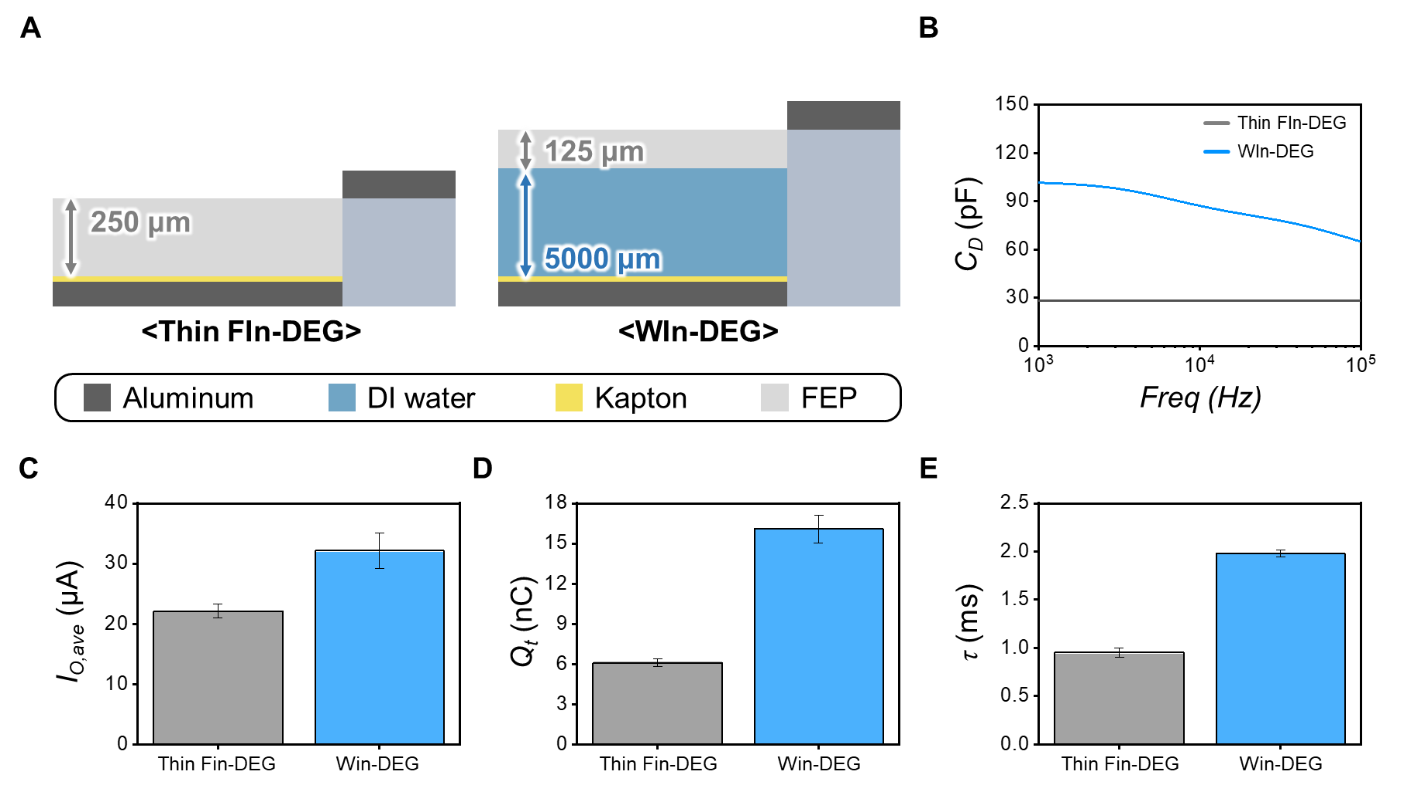
**

**Figure S4.** The electrical output comparison between the thin FIn-DEG similar to conventional DEG model and WIn-DEG. **A)** The schematic of the system composition between the thin FIn-DEG and WIn-DEG, and **B)** their $C_{D}$ behavior. **C)** $I_{O,ave}$ **D)** $Q_{t}$, and **E)** $\tau$ behavior under the thin FIn-DEG and WIn-DEG.

**
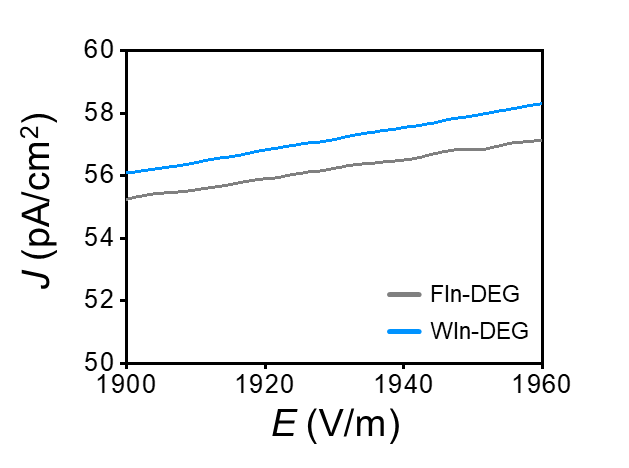
**

**Figure S5.** The leakage current density comparison between FIn-DEG and WIn-DEG.

**
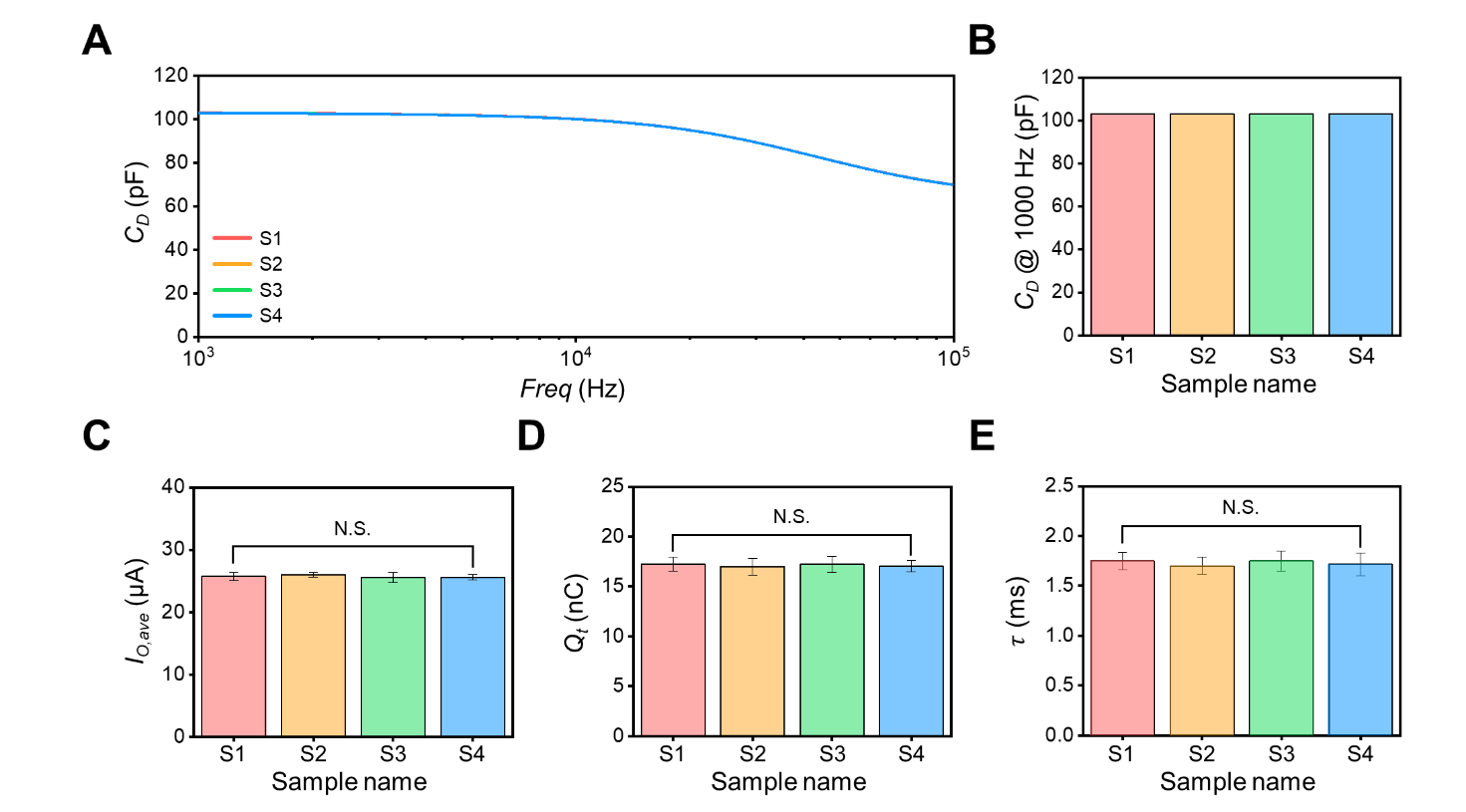
**

**Figure S6.** The device-to-device variation of the WIn-DEG. **A)** $C_{D}$ and **B)** $C_{D}$ at 1000 Hz under Sample 1 to Sample 4. **C)** $I_{O,ave}$ **D)** $Q_{t}$, and **E)** $\tau$ behavior under Sample 1 to Sample 4, and they are not statistically significant (with p < 0.05)

**
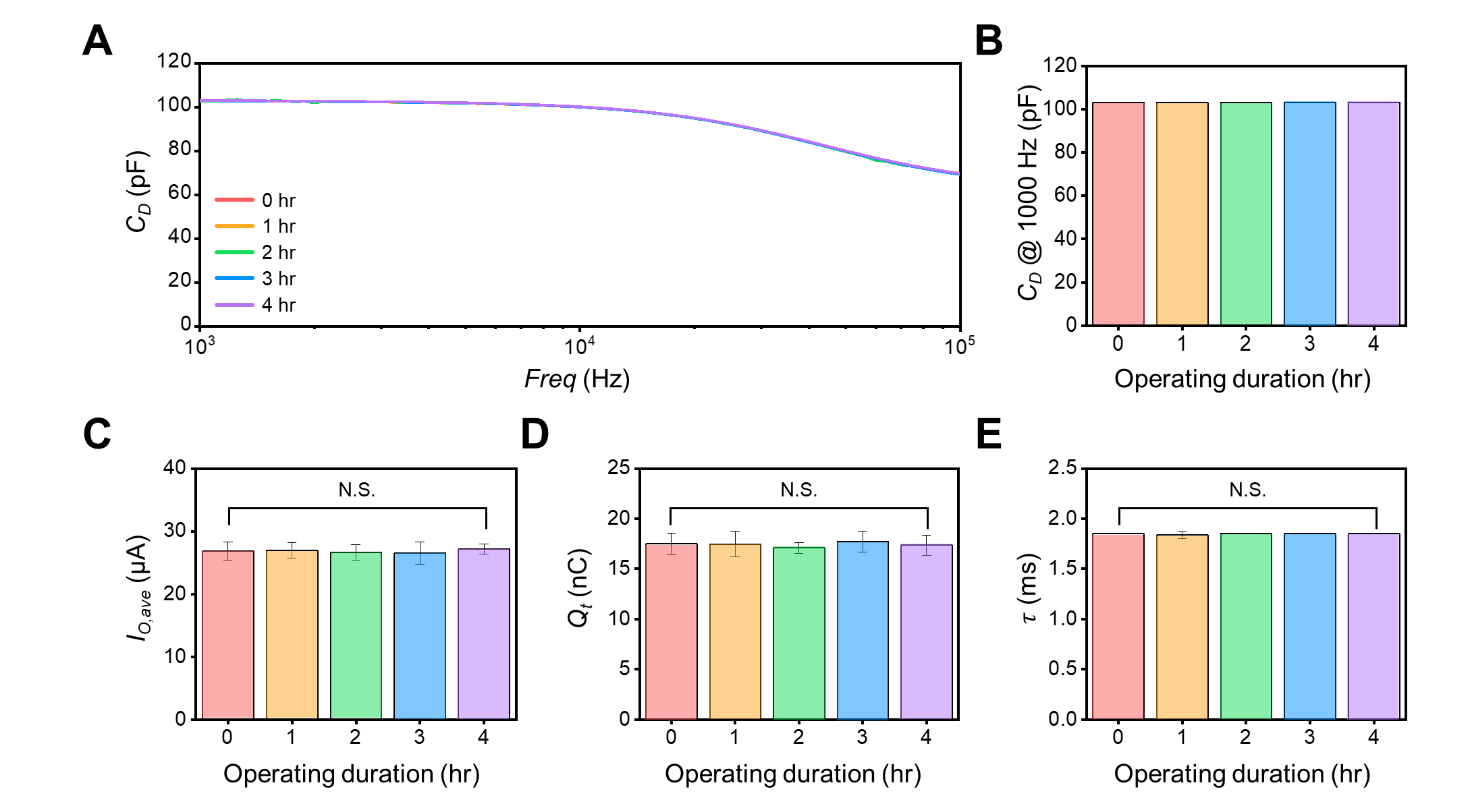
**

**Figure S7.** The stability of the WIn-DEG along 4 hour continuous operation. **A)** $C_{D}$ and **B)** $C_{D}$ at 1000 Hz under each operation hour. **C)** $I_{O,ave}$ **D)** $Q_{t}$, and **E)** $\tau$ behavior under each operation hour, and they are not statistically significant (with p < 0.05)


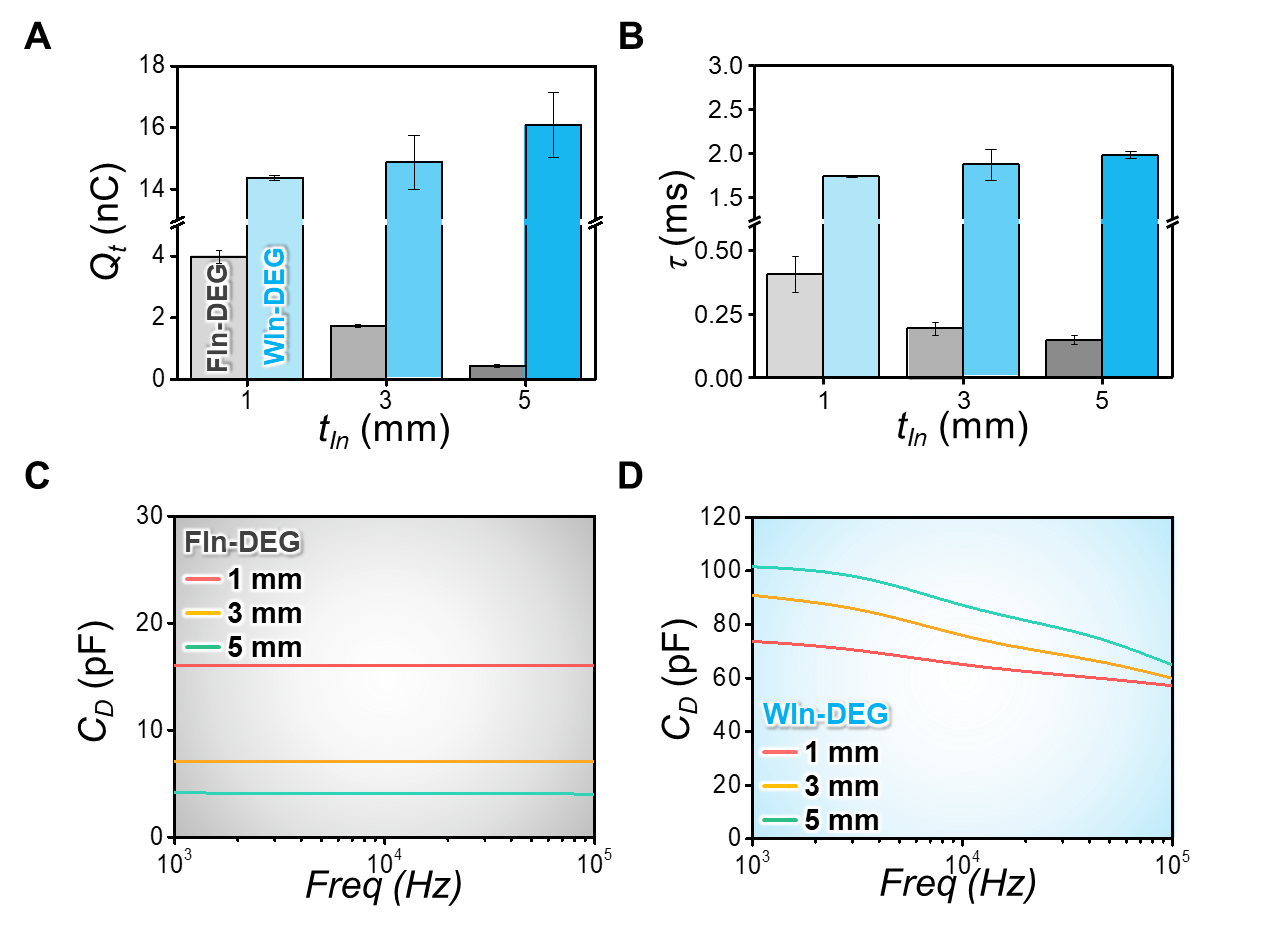


**Figure S8. A)** $Q_{t}$ and **B)** $\tau$ behavior under the various thickness of the intermediate layer ($t_{In}$). The $C_{D}$ of the **C)** FIn-DEG and **D)** WIn-DEG with various $t_{In}$.

**
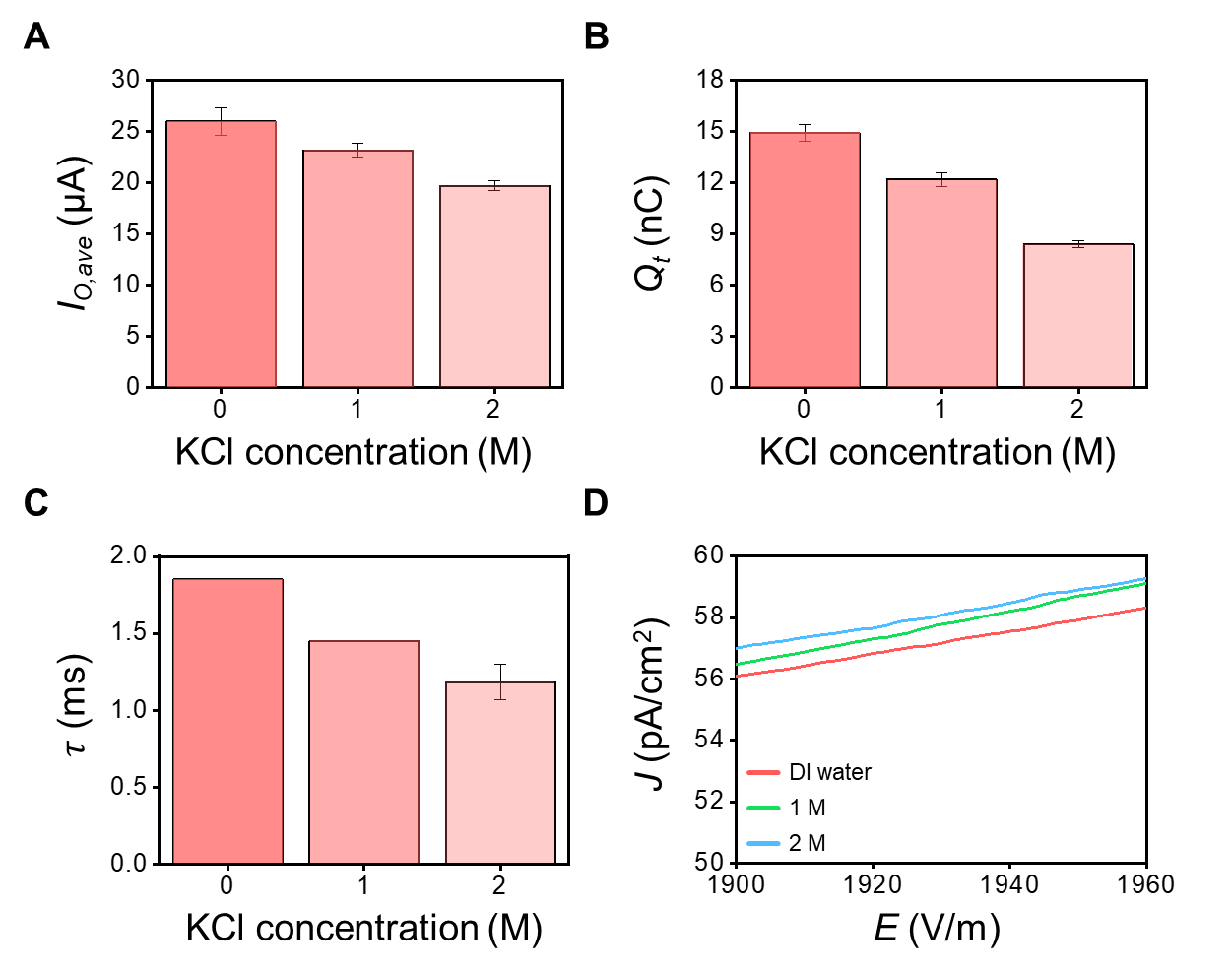
**

**Figure S9.** The WIn layer ionic composition effect under the WIn layer composition of DI water, 1 M and 2 M KCl aqueous solution. **A)** $I_{O,ave}$ **B)** $Q_{t}$, **C)** $\tau$ behavior, and **D)** leakage current density under each condition.


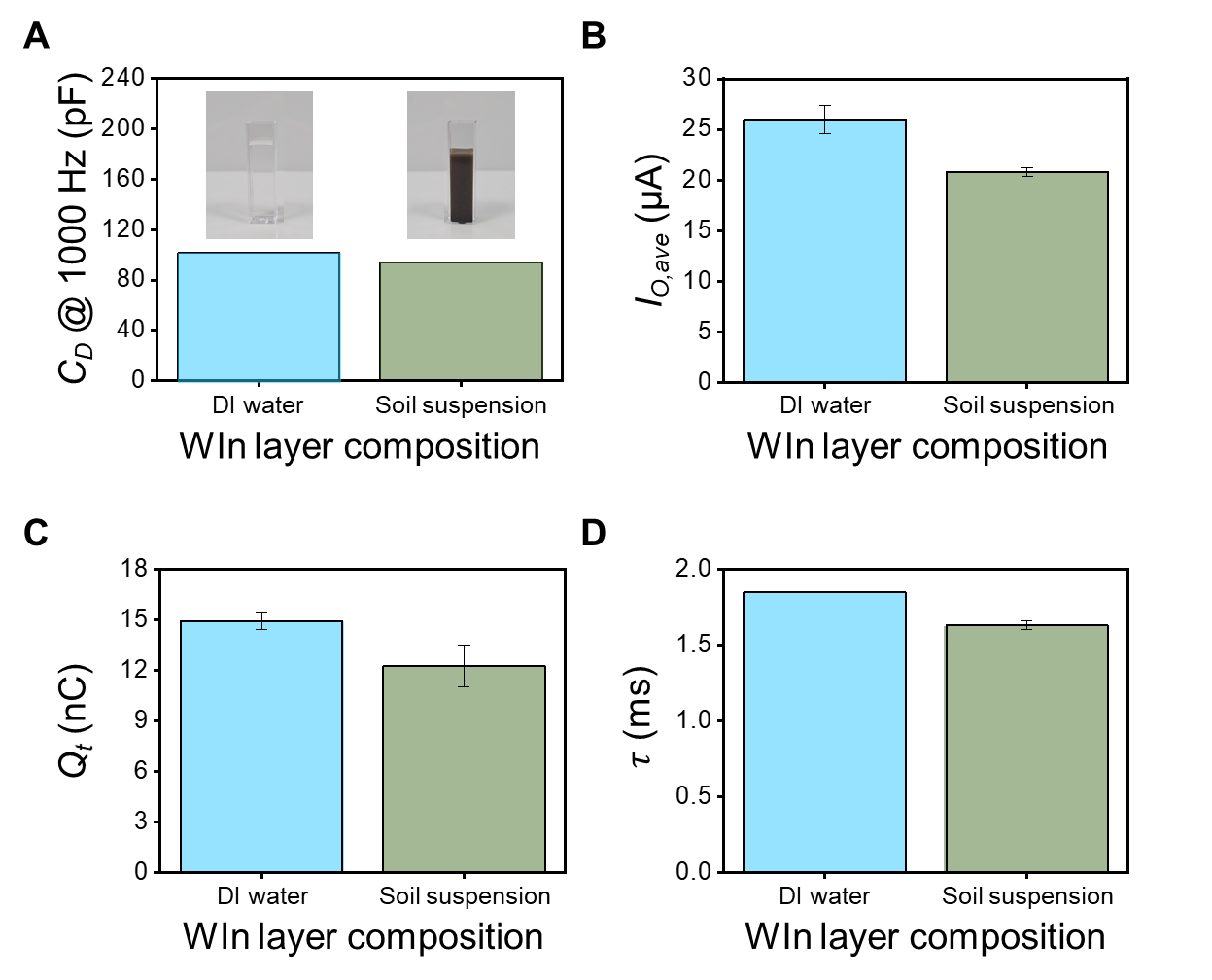


**Figure S10.** The WIn layer contamination effect under the WIn layer composition of DI water and soil suspension. **A)** $C_{D}$ at 1000 Hz, **B)** $Q_{t}$, **C)** $Q_{t}$, and **D)** $\tau$ behavior under each condition. The **inset in** **A)** shows the image of the DI water and soil suspension.


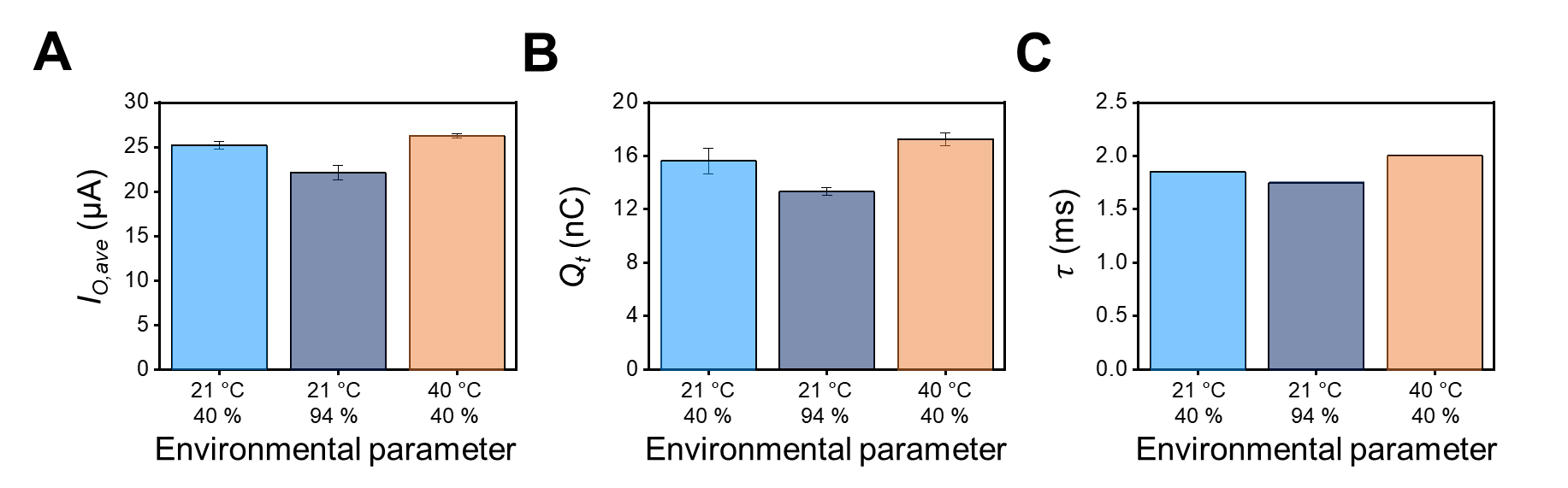


**Figure S11.** The environmental parameter effect on the WIn layer operation. **A)** $I_{O,ave}$ **B)** $Q_{t}$, and **C)** $\tau$ behavior, under the room condition (21 °C, RH 40 %), highly humid condition (21 °C, RH 94 %), and WIn layer heated condition (WIn layer temperature 40 °C, RH 40 %)


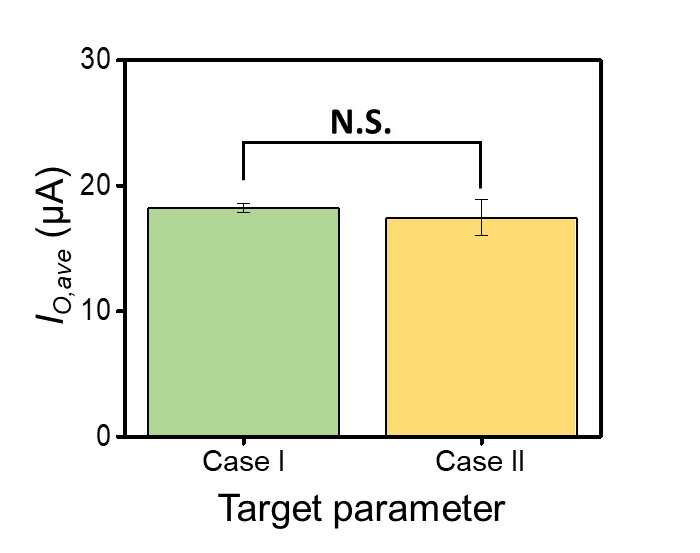


**Figure S12.** The $I_{O,ave}$ generated under the Case I and II. Based on the $I_{O,ave}$, they are not statistically significant (with p < 0.05).


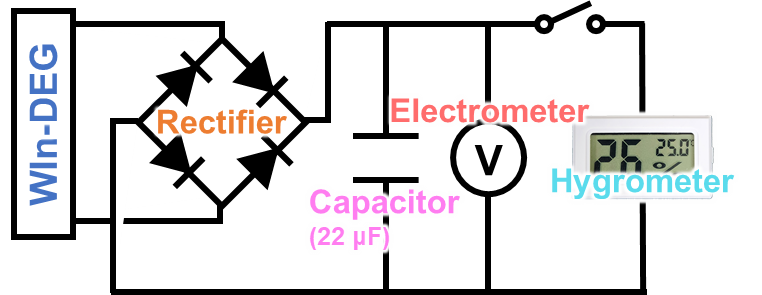


**Figure S13.** The circuit for the hygrometer operation with WIn-DEG.

**References**

[1] Wang, K., Xu, W., Li, J., Zheng, H., Sun, S., Song, W., ... & Wang, Z. Enhancing water droplet-based electricity generator by harnessing multiple-dielectric layers structure. *Nano Energy* **2023**, 111, 108388.

[2] Wang, H., Kurokawa, Y., Gotoh, K., Kato, S., Yamada, S., Itoh, T., & Usami, N. Performance enhancement of droplet-based electricity generator using a CYTOP intermediate layer. *Japanese Journal of Applied Physics* **2023**, 62(SC), SC1032.

[3] Zhou, Y., Zeng, Y., Wang, J., Li, X., Wang, P., Ma, W., ... & Zhang, D. Enhancement of the voltage output of droplet electricity generators using high dielectric high-entropy oxide composites. *Energy & Environmental Science* **2024**, 17(10), 3580-3593.

[4] Li, X., Ning, X., Li, L., Wang, X., Li, B., Li, J., ... & Guo, W. Performance and power management of droplets-based electricity generators. *Nano Energy* **2022**, 92, 106705.

[5] Liu, M., Ding, X., Xu, L., & Song, Y. Dual Enhancement in Output Voltage and Energy of Droplet Electricity Generator via a Composite Dielectric Layer Design. *Small* **2025**, e07038.

[6] Jang, S., Shah, S. A., Lee, J., Cho, S., Kam, D., Ra, Y., ... & Choi, D. Beyond metallic electrode: spontaneous formation of fluidic electrodes from operational liquid in highly functional droplet‐based electricity generator. *Advanced Materials* **2024**, 36(35), 2403090.

[7] Sriphan, S., Pharino, U., Chaithaweep, K., & Vittayakorn, N. Equivalent circuit model and simulation for dynamic sliding droplet-based triboelectric nanogenerators. *Nano Energy* **2024**, 130, 110100.

[8] Kam, D., Choi, H., Gwon, G., Jang, S., Shah, S. A., Yoo, D., & Choi, D. Toward Droplet Energy Harvesting in Harsh Environment: Mechanical Buckling‐Induced 3D Structured Droplet‐Based Electricity Multigenerator. *Small* **2025**, 21(25), 2502717.

[9] Nie, J., Ren, Z., Xu, L., Lin, S., Zhan, F., Chen, X., & Wang, Z. L. Probing contact‐electrification‐induced electron and ion transfers at a liquid–solid interface. *Advanced Materials* **2020**, 32(2), 1905696.
